# Metabolic Profiling of healthy and cancerous tissues in 2D and 3D

**DOI:** 10.1038/s41598-017-15325-5

**Published:** 2017-11-10

**Authors:** Shonagh Russell, Jonathan Wojtkowiak, Andy Neilson, Robert J. Gillies

**Affiliations:** 1 Department of Cancer Imaging and Metabolism, H Lee Moffitt Cancer Centre and Research Institute, Tampa, FL USA; 2 Agilent Technologies (Seahorse Bioscience), 5301 Stevens Creek Blvd., Santa Clara, CA 95051 USA; 3 Genentech Inc., 15 Pemberton Rd, Wayland, MA 01778 USA; 4 University of South Florida, Tampa, FL USA

## Abstract

Metabolism is a compartmentalized process, and it is apparent in studying cancer that tumors, like normal tissues, demonstrate metabolic cooperation between different cell types. Metabolic profiling of cells in 2D culture systems often fails to reflect the metabolism occurring within tissues *in vivo* due to lack of other cell types and 3D interaction. We designed a tooling and methodology to metabolically profile and compare 2D cultures with cancer cell spheroids, and microtissue slices from tumors, and normal organs. We observed differences in the basal metabolism of 2D and 3D cell cultures in response to metabolic inhibitors, and chemotherapeutics. The metabolic profiles of microtissues derived from normal organs (heart, kidney) were relatively consistent when comparing microtissues derived from the same organ. Treatment of heart and kidney microtissues with cardio- or nephro-toxins had early and marked effects on tissue metabolism. In contrast, microtissues derived from different regions of the same tumors exhibited significant metabolic heterogeneity, which correlated to histology. Hence, metabolic profiling of complex microtissues is necessary to understand the effects of metabolic co-operation and how this interaction, not only can be targeted for treatment, but this method can be used as a reproducible, early and sensitive measure of drug toxicity.

## Introduction

From the time of Cori and Cori^1^, it has been understood that some cells generate metabolic “waste”, sometimes at a distance, which is subsequently consumed by other cells. Tissues commonly exhibit inter- and intra-organ metabolic co-operation. For example, during periods of starvation: the liver produces ketone bodies to fuel the brain^2^; skeletal muscle produces lactate which the liver converts into glucose^3^; glia cells in the central nervous system produce lactate, consumed by neurons^4^.

It has been recently appreciated that tumors have evolved metabolic cooperation wherein fermentative cells consume glucose to produce lactate, and oxidative cells consume lactate for respiration^5,6^. Tumor survival is based on its ability to adapt to dynamic changes, such as, pH^7^, reactive oxygen species (ROS)^8^, nutrient supplies^9^ and hypoxia^10^, all of which can exert evolutionary selective pressure. Adaptations to these factors generate phenotypic and genotypic heterogeneity, which is a proximal cause of therapy resistance^11^. Successful targeting of cancer is therefore a daunting task due to metabolic, genomic and physiological heterogeneity. We contend that assessment of metabolic responses in complex tissues provides a drug testing paradigm that accounts for such complexity and, perhaps, can improve the success rates in screening of new drug candidates, especially emerging therapies targeted to metabolic disruption^12,13^.

2D monolayers fail to recapitulate the 3D interactions harbored within a tumor, including the effect of cell: cell interaction^14^, nutrient gradients and the role of microenvironmental stress in 3D, as opposed to 2D, models^15^. This may have bearing on the failure of agents to succeed *in vivo* after showing promise in 2D monolayer culture. In recent years, the technology to produce 3D cell culture models has improved^16,17^, enabling semi high-throughput, reliable production of 3D spheroids from multiple different cell types^18^. As a counterpoint to drug efficacy, off-target toxicity is a major hurdle for the clinic and is a primary endpoint in phase I clinical trials. Cardiac and nephro- toxicities are common limitations and are often not observed until completion of rigorous toxicity testing or, in some cases, during expanded cohorts in phase II or phase III clinical trials^19^.

In cancer, therapeutics commonly affect tumor and stroma cellular metabolism, either directly or indirectly^20^.The Warburg effect and “reverse Warburg effect”^21^ are examples of metabolic plasticity^22^ that are observed frequently in cancer, enabling a constant fitness advantage regardless of the environmental constraints. High throughput metabolic profiling using, e.g. the Seahorse Bioscience extracellular flux (XF) analyzer has enabled observation of differences between normal and cancerous cell lines, effects of microenvironmental stress and the ability of drugs to alter the metabolic phenotypes of a 2D cell culture monolayer^23–25^. Further, cytotoxic perturbations in metabolism are often observed prior to cell death^26^ and hence, metabolic profiling can be a key data set in drug development. However, until now, there has been no high-throughput, reliable method for studying metabolism of 3D culture or complex microtissues in comparison to 2D monolayer cultures.

In this study, we developed a micro-chamber system designed to enable metabolic profiling 3D spheroid cultures and microtissues from normal organs and tumors. These data were compared to metabolic profiles obtained from 2D monolayers. Subsequently, this method was able to be utilized in multiple cell lines, tumors and organ types in a moderately high throughput manner and differential effects of chemotherapeutics on 2D 3D cell cultures and microtissues were observed. This technique can be used to further basic science and understanding of differences in 2D and 3D models and utilized as a key step for efficacy and toxicity testing prior to *in vivo* studies or clinical trials.

## Results

### Metabolic Profiling of a 3D Culture

**Figure 1:**
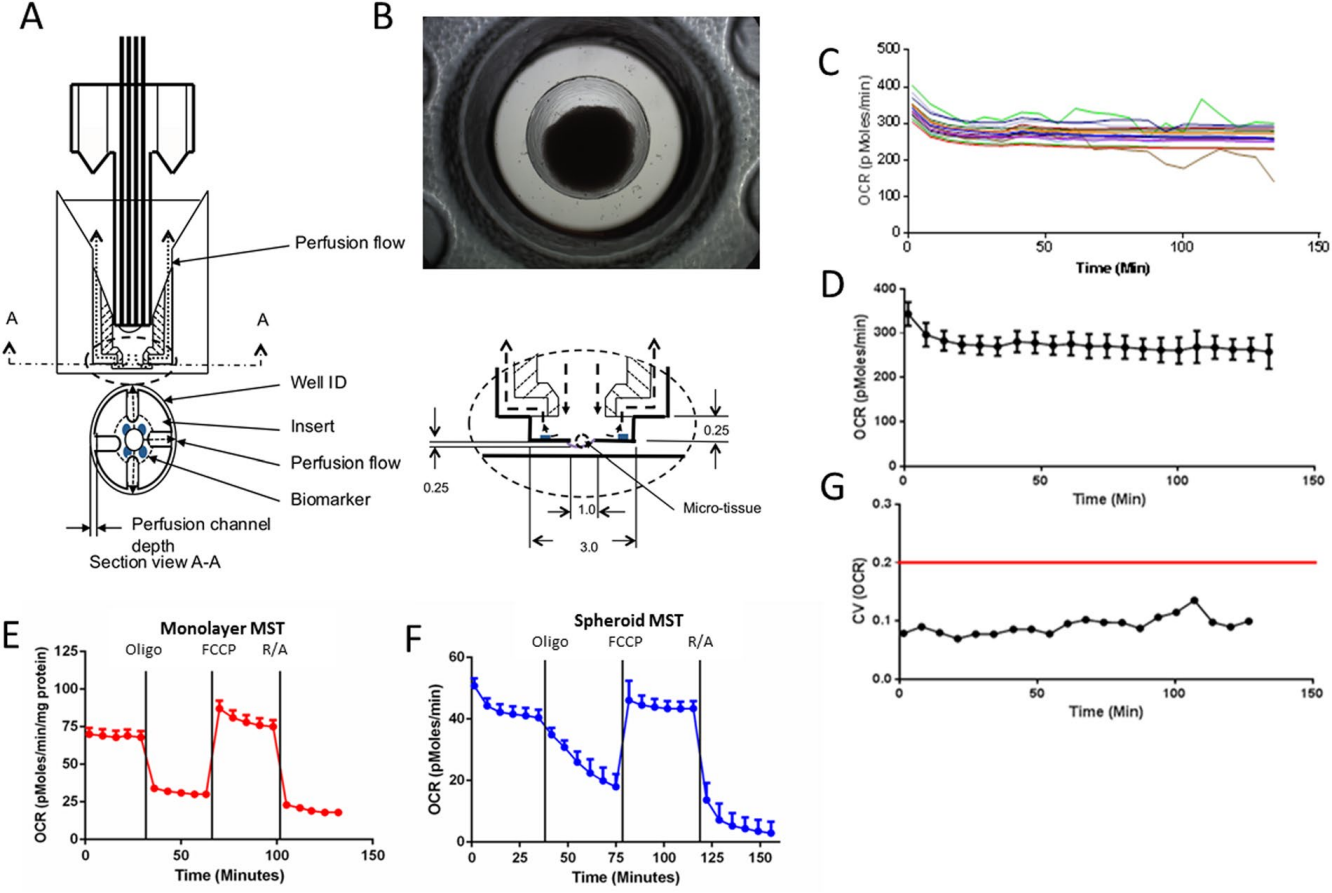
Metabolic Profiling of 2D vs. 3D cell culture model. (**1A**) Schematic of the microtissue perfusion insert placed in each well of a seahorse flux pack to enable localization of a spheroid during metabolic profiling. (**B**) Representative image (4X magnification) of an HCT116 spheroid within a microtissue perfusion insert. Average diameter 829.332 ± 27.13 µM. (**C**) OCR of HCT116 spheroids over time plotted individually (n = 16). (**D**) Oxygen Consumption Rate (OCR) of the spheroids as a group, OCR is maintained over a period of 140 mins. (**E**) Mitochondrial stress test in an HCT116 colorectal cancer 2D monolayer 12.5k cells per well, undertaken using Seahorse XFe96 Extracellular Flux Analyzer. Oligomycin (1 uM), FCCP(1 uM) and Rotenone/Antimycin A(1 uM) used to test mitochondrial metabolism. (**F**) Mitochondrial stress test in an HCT116 colorectal cancer 3D spheroid model. G: Coefficient of variation <20% within the spheroid OCR values observed. Error bars represent + -SEM.

To directly compare metabolic phenotype between 2D and 3D cultures, we developed a tool allowing 3D profiling in the same technology used for 2D monolayer cultures- the Agilent Seahorse XFe96 Flux Analyzer, in a 96-well plate format. The tooling design (Fig. 1A) enables a spheroid or microtissue to sit within an indent in a single well of the 96-well plates (Fig. 1B), preventing movement and allowing the production of a micro-chamber to measure both oxygen consumption rate (OCR) and extracellular acidification rate (ECAR). This micro-chamber formation^27^ mimics the conditions a monolayer undergoes when being metabolically phenotyped.

We generated HCT116 spheroids using the hanging droplet method^28^. Across a population of HCT116 spheroids, there was a minimal dispersion in the oxygen consumption rate (OCR) (Fig. 1C). When we grouped the spheroids, we observed minimal variation, suggesting that there was a low level of inter-spheroid metabolic heterogeneity (Fig. 1D). Statistical analysis determined that the coefficient of variance (CoV) was < 10% (Fig. 1G), which allows group analysis and comparison to 2D monolayers. This assay also showed the designed tooling (Fig. 1A) was able to centralize the spheroid with minimal movement as can be seen by the steady OCR/ECAR values (Fig. 1C). Notably, movement of a spheroid within the tooling can occur as the plunger goes up and down but is detectable by a transient increase or decrease in OCR, e.g., Fig. 1C at 100 and 110 minutes.

After verifying that the tooling was able to be utilized for metabolic profiling of the 3D spheroid cultures, we performed an Agilent Seahorse XF Cell Mitochondrial Stress Test (MST) to determine if there were differences metabolically between 2D and 3D cultures in both basal metabolism and response to metabolic inhibitors. The MST assay consists of sequential additions of oligomycin to inhibit the ATP synthase^29^, FCCP, an uncoupler that induces maximal OCR, and a combination of Rotenone/Antimycin A to block the electron transport chain revealing the extent of non-mitochondrial oxygen consumption. Figure 1E and F show a direct comparison of mitochondrial respiration from multiple wells containing 2D (Figs 1E and 3D (Fig. 1F) cultures of HCT116 colon cancer cells. Notably, in both systems, the inter-sample variability was low, as evidenced by the error bars (S.E.M.). In the 3D model, we observed delayed effects upon addition of oligomycin, but not with FCCP or R/A. We initially attributed this to the molecular weight of oligomycin (~791, logP = 5.88) and an associated retarded penetration into the core of the spheroid compared to a 2D monolayer. However, use of similar ATP synthase inhibitor, N, N-dicyclohexylcarbodiimide (DCCD) with a lower molecular weight and similar polarity (~206, logP = 5.54) induced the same delayed inhibition of oxygen consumption (data not shown); suggesting that penetration alone was not responsible for the delayed effect. Therefore, reduced sensitivity to the ATP synthase may be an intrinsic characteristic of the 3D culture. This particular difference in response to a common metabolic inhibitor leads us to believe that there would be observable differences in the metabolic profile of 2D and 3D cultures in response to different chemotherapeutics.

**Figure 2:**
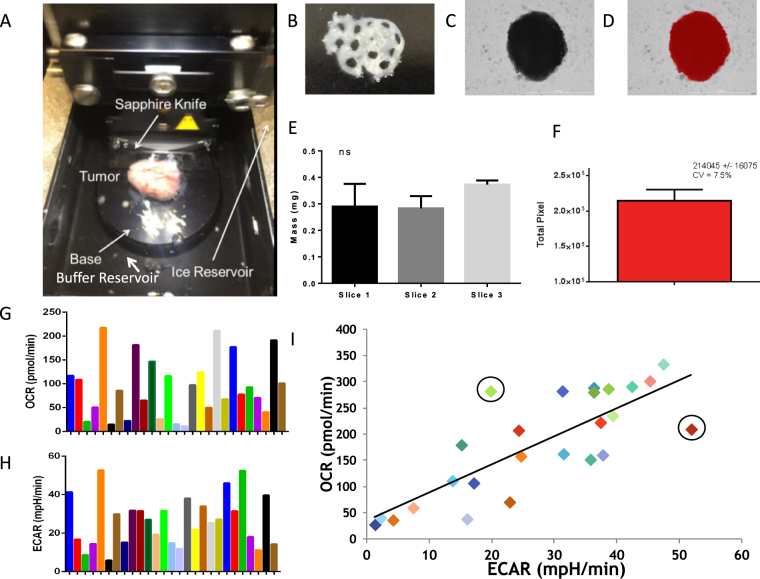
Generation of microtissues from human xenograft HCT116 tumors and MIA PaCa-2 tumors. (**A**) Microtissue production image using a vibratome with a sapphire knife to generate tumor slices for microtissue generation using a 1 mm unicore punch. (**B**) HCT116 tissue slice (0.5 uM thick) with microtissues harvested using 1 mm Harris Uni-core punch. (**C**) HCT 116 Spheroid (**D**) Definiens software border detection analysis of HCT 116 spheroid. (**E**) Average mass of microtissues (n = 3) from 3 tumor slices, no significant difference. (**F**) Definiens total pixel count analysis of microtissue, n = 3 CV 7.5% SD ns. (**G**) Average OCR of MIA PaCa-2 tumor derived microtissues (n = 26). (**H**) Corresponding ECAR of each microtissue from Fig. 3G. (**I**) Scatter plot of OCR vs ECAR of each microtissue, a linear trend is observed but not significant.

### Metabolic Profiling of Tumor Derived Microtissues

We investigated the generation of microtissues from human cell line tumor xenografts in a high-throughput manner similar to spheroids. First, we had to determine if it was possible to produce microtissues from excised tumors in a reproducible manner. This procedure required minimal damage occurring to the microtissue to enable it to be maintained alive *ex vivo* for a period sufficient to carry out metabolic profiling. Microtissues were generated from 0.5 mm slices from MiaPaca2 tumors (Fig. 2A). In the case presented, three different slices were generated and, from these, 1 mm diameter microtissues were obtained from these slices using a Harris trochar punch (Fig. 2B). The sizes of these tissues were measured physically by mass and computationally by imaging. Nine individual microtissues (generated from three different slices) were weighed individually and showed no statistically significant differences in mass (Fig. 2E). An alternative method to determine the size of the microtissue was quantifying the number of pixels within the computationally identified border of the spheroid or microtissue (Fig. 2C and D). This method produced a coefficient of variation of 7.5% (Fig. 2F). Therefore the two methods of measuring mass showed the microtissues could be produced reliably with negligible size variation. MiaPaCa-2 microtissues were profiled for over 24 hours before seeing a metabolic decline (Fig. S2A and B). On the other hand, Lewis lung carcinoma (LL2) microtissues exhibited no metabolic decline up to 30 hours *ex vivo* (Fig. S2C and D), suggesting genetic and phenotypic diversity can influence microtissue survival and suggests there may also be disparities in response to a chemotherapeutic challenge.

We examined the metabolic profile of microtissues generated from MiaPaca2 and HCT 116 tumors. Measuring both the basal OCR and glucose-induced ECAR of each microtissue demonstrated a large amount of heterogeneity between different microtissues captured from the same tumors, despite similarities in microtissue and tumor size. Figure 2G and H show representative heterogeneity of OCR and ECAR from 26 different microtissues from a single MiaPaCa2 tumor. This heterogeneity is expected as there are multiple regions (habitats) within tumors that contain different sub-populations of cells, including metabolically different cancer cells and contributions from stromal fibroblasts, endothelial cells, smooth muscle cells, immune cells and necrosis^30,31^. Despite the heterogeneity apparent in Fig. 2G and H, plotting the OCR vs. ECAR for each microtissue (Fig. 2I) showed a positive correlation (R^2^ = 0.642), which suggests similar metabolic profiles across the tumor and that increased values corresponded to increases in metabolically active tissue. Although there was a high correlation, there were some apparent “outliers” exhibiting abnormally high or low OCR/ECAR ratios (Fig. 2I circles). The cause and identity of these outliers have yet to be determined. Nonetheless, such variations underscore the complexity of tissue architecture and why tumor targeting can be such a challenge. Tumor heterogeneity increases with increasing tumor size and, to see if we also observed this metabolically, we generated microtissues from different MiaPaCa-2 tumors of ca. 400 mm^3^ and 2000 mm^3^ in volume. Supplemental Figures 12A and 12B show the OCR for each microtissue as a function of time and indicated a higher amount of dispersion in OCR and a much lower metabolic output from the microtissues obtained from the larger, compared to the smaller, tumors. Such an observation would be consistent with increased volumes of necrosis and avasculature within larger tumors, resulting in reduced metabolic output.

**Figure 3:**
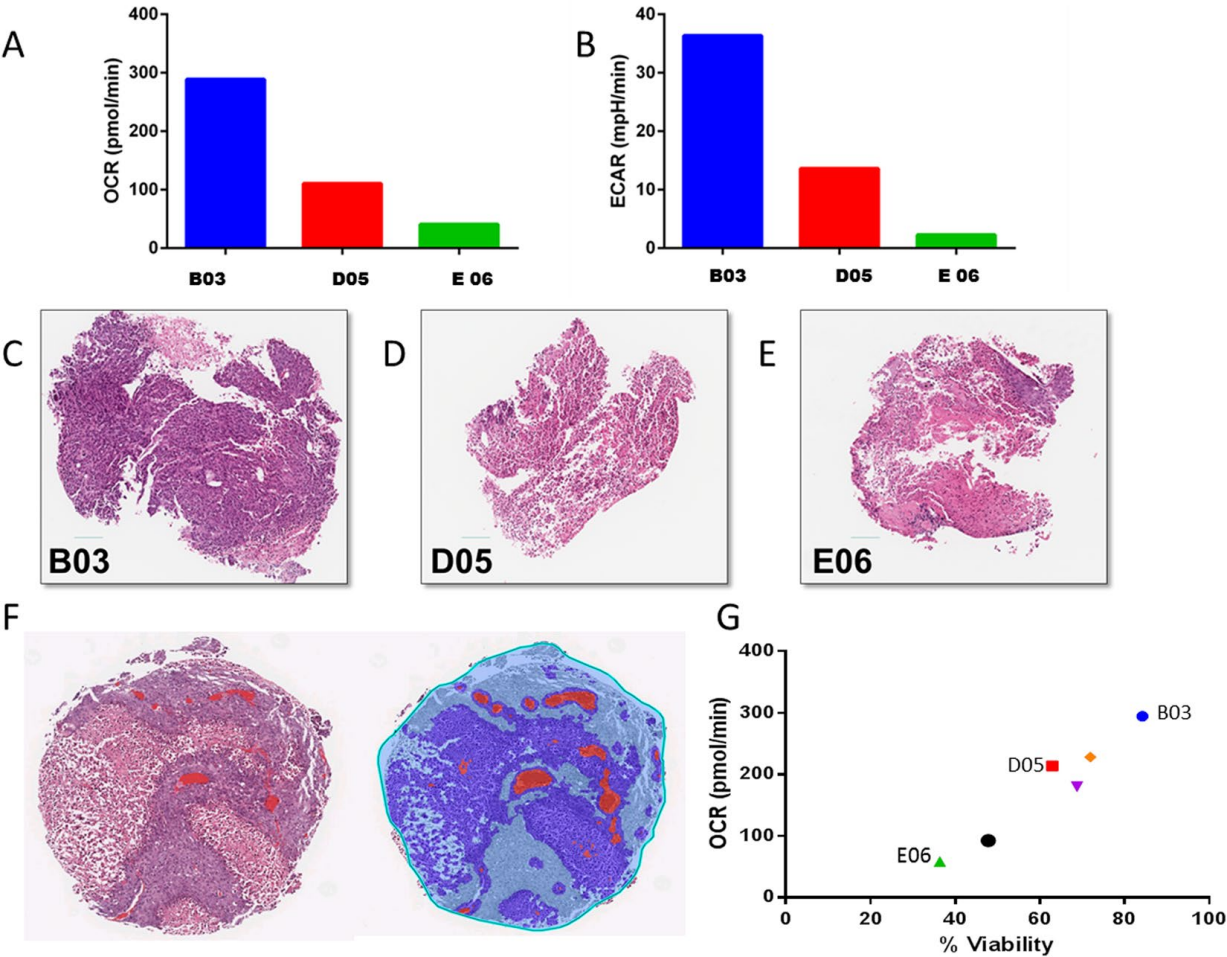
Microtissue Viability Correlates to Metabolic Phenotype. (**A**) Oxygen Consumption Rate (OCR) of three individual HCT116 derived tumor microtissues. (**B**) ECAR of three individual HCT116 derived tumor microtissues. (**C**–**E**) Hematoxylin and Eosin staining of tumor microtissue slices. (**F**) Digital Quantification of necrotic regions. (**G**) Positive Correlation of percent viable tissue from H and E staining with metabolic oxygen consumption rate, n = 6.

To investigate whether metabolic output correlated with tumor architecture, we compared metabolic profiles with histopathology from the same samples. We chose six different microtissues from the same MiaPaCa-2 tumor with a range of OCR and ECAR values. Figure 3A and B show representative values from 3 such microtissues. Following profiling, the tissues were fixed, paraffin embedded and the sections were stained with Hematoxylin and Eosin (H&E), shown in Fig. 3C–E, and for HCT116 microtissues shown in Supplemental Figures S1A–F. Necrotic areas were automatically scored, as in Fig. 3F, as unstained areas and the remaining stained areas, were quantified as percent viability of the microtissue. We correlated percent viability values to the OCR and, as expected, those microtissues with a reduced OCR had a reduced percent viability, for example, E06 (Fig. 3E,G). Conversely, microtissues with higher OCR had an increased percent viability, for example, B03 (Fig. 3C,G). Based on these observations, we believe that, not only can these microtissues be metabolically profiled but also that the metabolic value can be related phenotypically and genotypically to specific microtissues, formulating a more informed profile of metabolic heterogeneity and the habitats within each tumor.

### Chemotherapeutics on 2D vs. 3D cell culture models

**Figure 4:**
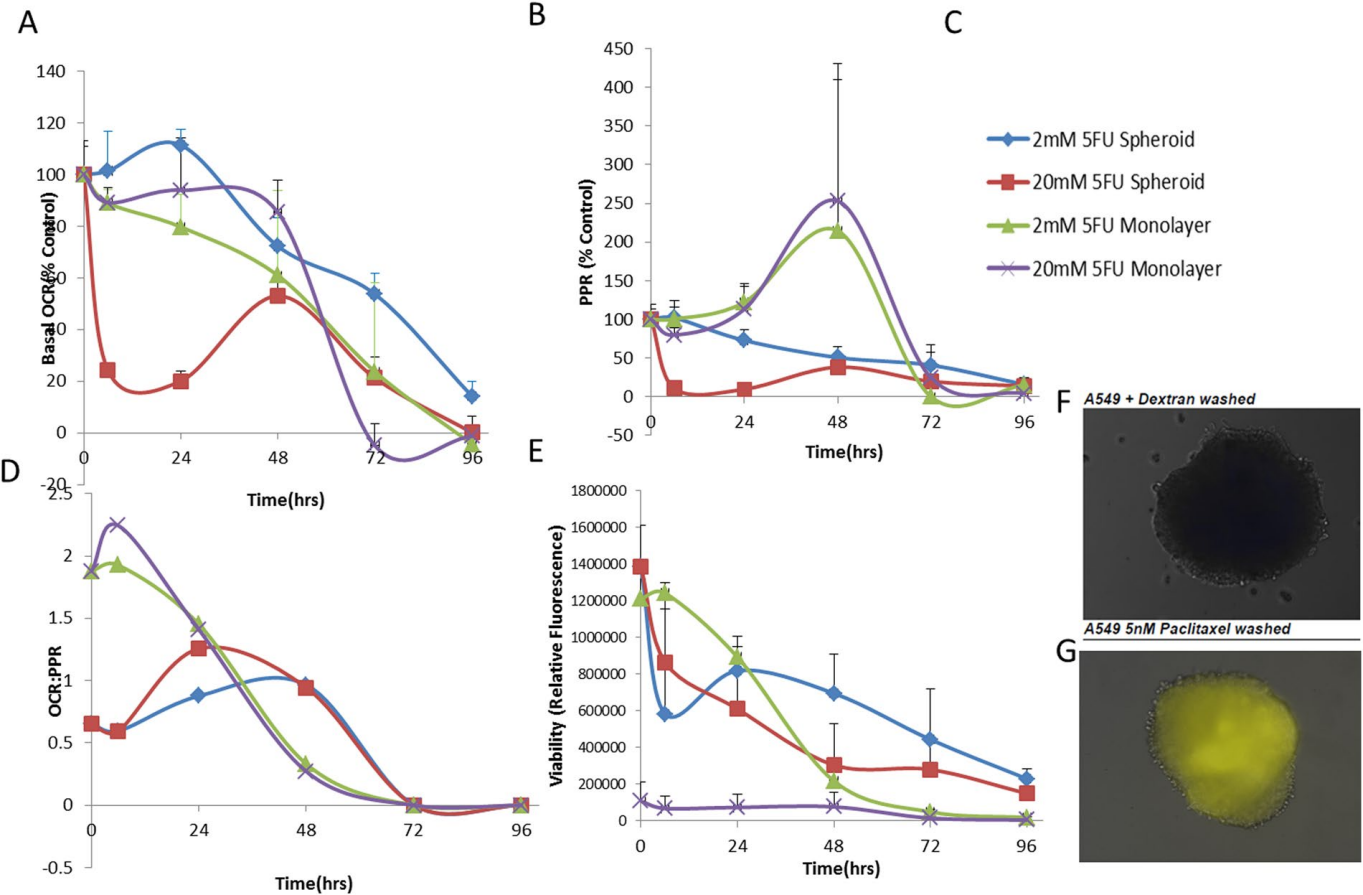
Effects of 5FU on 3D spheroid metabolic phenotype versus 2D monolayer in HCT116 colorectal cancer cell line. (**A**) Basal OCR as a percent of control (untreated 2D or 3D) upon treatment with 2 or 20 mM 5-Fluorouracil in 2D or 3D cell culture model. (**B**) Basal ECAR as a percent of control (untreated 2D or 3D) upon treatment with 2 or 20 mM 5-Fluorouracil in 2D or 3D cell culture model. (**C**) Group Key for Panel A,B,D,E. (**D**) OCR:PPR ratio of 2D or 3D cell model with 5FU treatment (2 or 20 mM). (**E**) Percentage viability of cells over time in the 2D or 3D model upon exposure to 2 or 20 mM 5FU using Cell Titer Glo assay. (**F**) A549 spheroid incubated with fluorescent dextran after washing. (**G**) A549 spheroid treated with fluorescent dextran and 5 nM paclitaxel (pseudo-color yellow).

**Figure 5:**
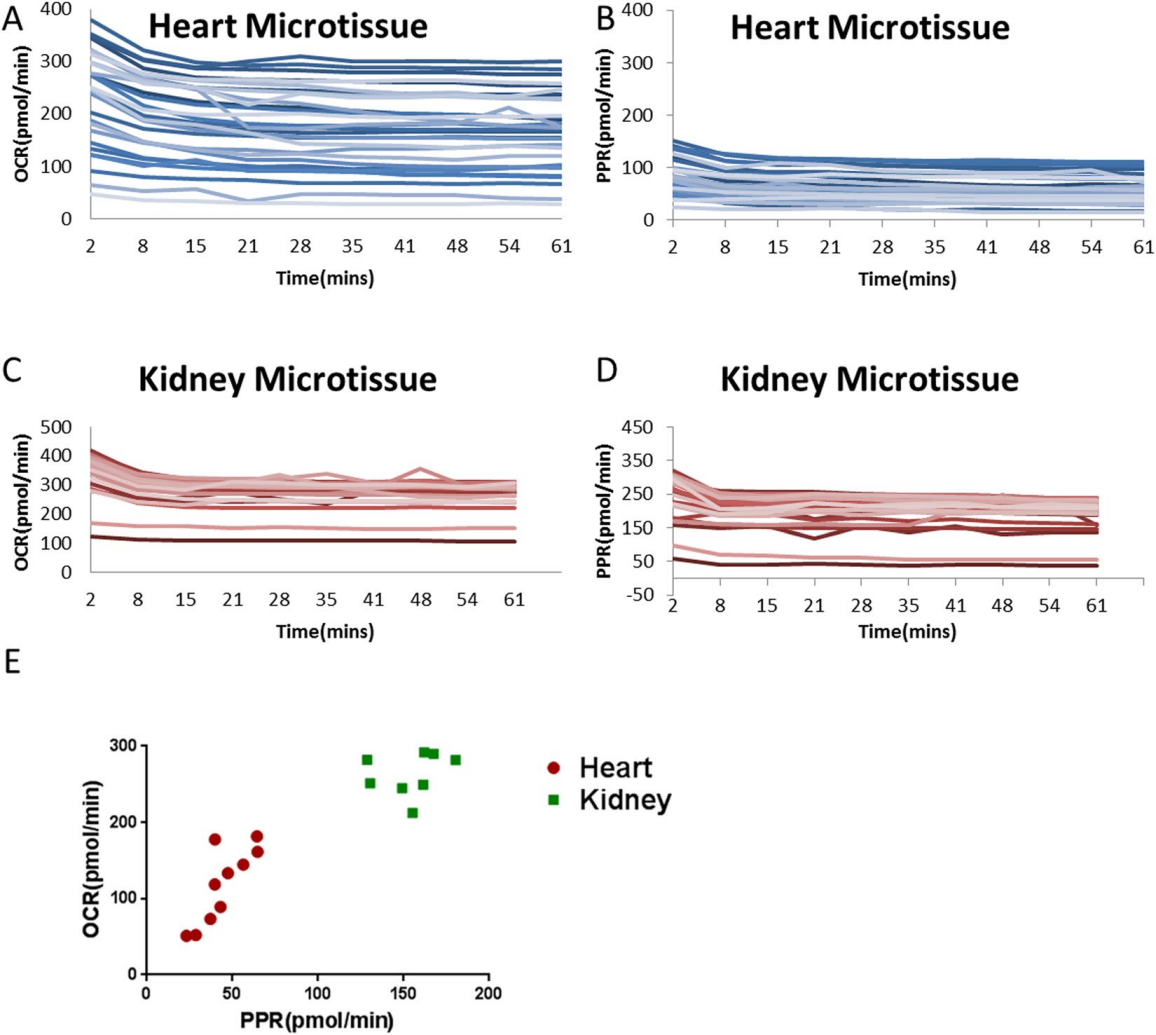
Profiling of microtissues from organs. (**A**,**B**) OCR and PPR of mouse heart microtissues generated using the same method for producing tumor microtissues, profiled over 60 minutes (n = 30). (**C**,**D**) OCR and PPR of mouse kidney microtissues profiled over 60 minutes (n = 30). (**E**) Average OCR vs PPR of heart and kidney microtissues after 60 minutes (n = 10 per group).

In drug development, attrition rates increase when transitioning from *in vitro* to *in vivo* models. James Galle – Pharmacoknetic driven drug development mt Sinai j med 2010 Hence, we studied whether we could observe any differences between 2D and 3D metabolic phenotypes when exposed to the same chemotherapeutic agents. 5-Fluorouracil (5-FU)^32^, a standard chemotherapeutic, was chosen and its effect on metabolism was profiled over 96 hours in both 2D and 3D models at 2 and 20 mM concentrations. In all cases, at both doses, 5-FU eventually resulted in complete metabolic inhibition, with disruption to metabolic phenotype between monolayer and spheroid (Fig. S5). Notably, 3D spheroids exhibited a lower basal level of PPR compared to the monolayer (Fig. 4B) and 5-FU had a delayed effect on OCR in 3D culture compared to 2D monolayer culture (Fig. 4A, Fig. S4A and B). The monolayer was much more oxidative than glycolytic at earlier time-points (Fig. 4D), and this started to revert after the 48-hour time point, suggesting 2D becomes metabolically stressed and energy stifled resulting in a switch to glycolysis to try and maintain energy production. Spheroids showed a more linear response to 5FU compared to the monolayer (Fig S5G). 5-FU disrupted the outer layer of the spheroids over time (Fig. S3A) and reduced spheroid density seen by H & E staining (Fig. S3B and C), but the removal of 5-FU did not revert the cellular edge disruption (data not shown).

Monolayer cultures all exhibited a spike of increased glycolytic activity at 48 hr. (Fig. 4B), which was reproducible and we suspect due to the increased energy and macromolecular synthesis requirements needed to survive chemotherapeutic exposure. In 3D culture, the PPR declined monotonically throughout the entire exposure period. OCR decreased more rapidly in the monolayer (Fig. 4A) as compared to the 3D spheroids under the same conditions in a parallel experiment, and this decrease is mirrored in cell viability of the monolayer(Fig. 4E). Notably, even at the end of the exposure period, spheroids remained metabolically viable throughout the 96 hour time course, determined with metabolic profiling and Cell Titer Glo, whereas the monolayers were dead by 96-hours, showing monolayers are more sensitive to 5FU (Fig. S4C and D). Other chemotherapeutics (rapamycin (Fig. S6), foretanib (Fig. S7), cisplatin (Fig. S10) and pemetrexed (Figs S8, S9 and S11)) were also tested and we observed variations in metabolism between the 2D and 3D models. Pemetrexed, in particular, has had disappointing efficacy in clinical trials^33,34^. In our studies, pemetrexed was effective in perturbing A549 monolayer metabolic output but not that of spheroids (Fig. S8A,C,D). We hypothesized that this insensitivity might be due to penetration of the drug into the spheroid, as its hydrated mass is ca. 1000 and its logP is ca. −1.5^35^. To investigate spheroid penetration, we employed paclitaxel, a microtubule-disrupting drug as an agent to disrupt cell: cell adhesions and theoretically promote penetration. We incubated spheroids in the presence of cascade blue dextran (MW~10 000; logP < −3) in the presence or absence of paclitaxel to see if we could improve penetration into the spheroid. Without paclitaxel, we observed no dextran in the spheroid (Fig. 4F), but in the presence of paclitaxel, dextran could penetrate all the way to the core (Fig. 4G). This observation suggested that penetration of hydrophilic kilo Dalton-sized agents into spheroids is hampered and can be promoted by paclitaxel. However, when metabolically profiled, the use of low concentration of paclitaxel to improve penetration, along with pemetrexed did not alter the metabolic phenotype (Fig. S9). Collectively, this suggests there are additional mechanisms that also play a role in altered drug efficacy^36^ of 3D cultures, for example, upregulation of pumps to export drugs such as Pgp, which is often exhibited in multicellular resistance, or downregulation of transporters to prevent drug uptake^37^.

### Organ Microtissue Metabolic Profiling

Finally, a major concern in drug development is toxicity to organs, such as cardio-^38^ or nephron-toxicity^39^. If drug toxicity is too high in patients, it can cause drugs to fail in clinical trials that have cost millions, if not billions, to get to that stage. Although this is something that is supposed to be seen in *in vivo* models and Phase 1 trials, we feel there should be additional steps taken to try and circumvent this potential toxicity in organs. To examine toxicity *ex vivo*, we generated organ microtissues in the same manner as tumor microtissues and profiled their metabolism. Individual heart microtissues were generated from the left and right ventricle and showed high variance in OCR (Fig. 5A), which may be due to differential cardiac myocyte populations within the ventricles. Furthermore, it has been shown previously that calcium flux can vary between adjacent cells in the heart^40^. Figure 5B shows that the PPR of heart microtissues is less varied than OCR. In contrast to heart microtissues, those derived from kidneys were primarily from the cortex and exhibited a low amount of inter-tissue variability (Fig. 5C and D). Figure 5E shows that overall the kidney is more metabolically active than heart tissues *ex vivo* and this may relate to the inability of the cardiac tissue to contract once excised as microtissues. Overall, the ability to profile organs, as well as tumor microtissues, could ensure not only efficacy but detection of toxic off-target effects. However, especially for heart tissues, for each measurement to be meaningful, each tissue must serve as its pre-treatment control. Hence, we took metabolic readings pre and post treatment with a drug.

**Figure 6:**
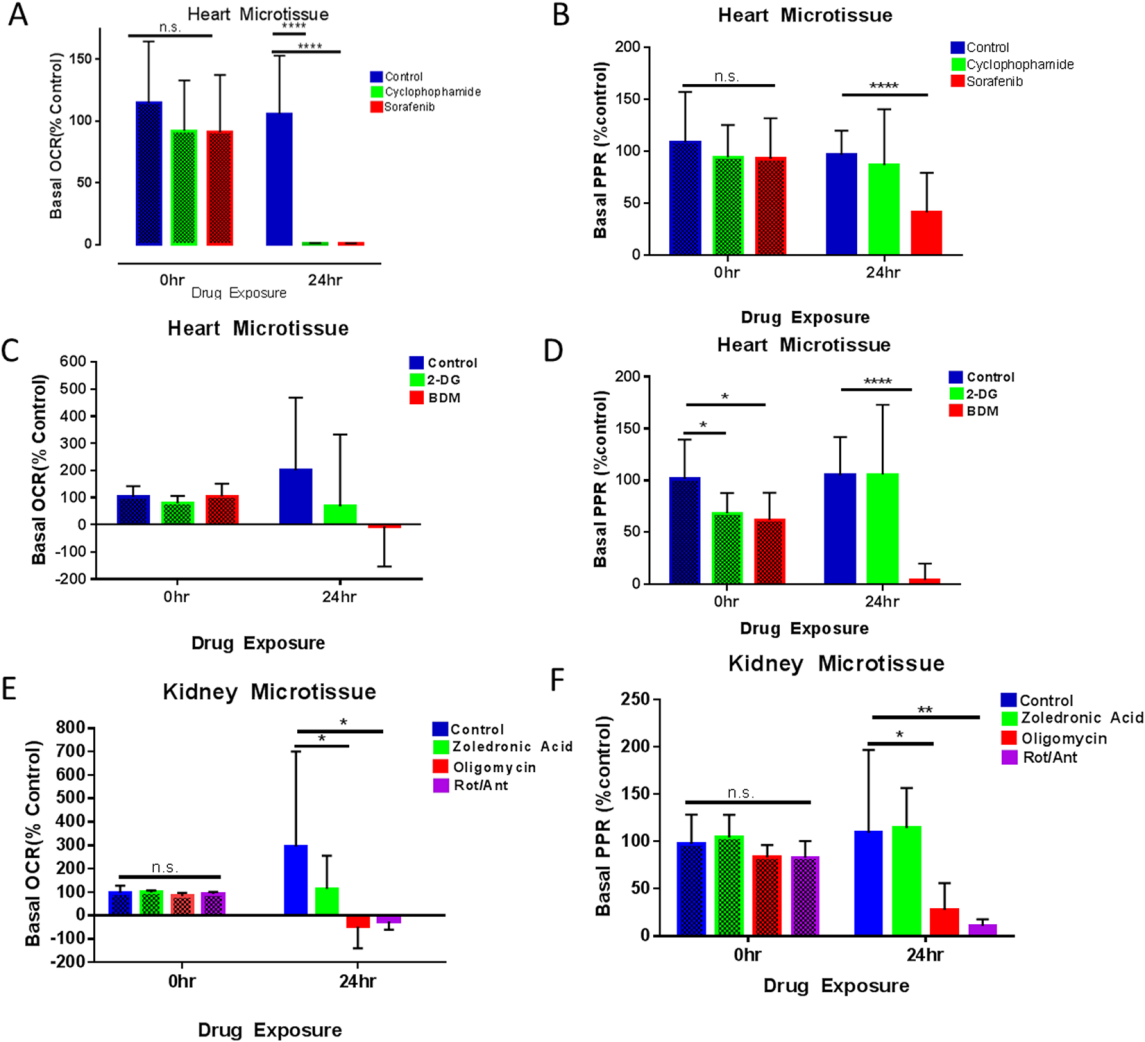
Toxicity studies in mouse heart and kidney microtissues. (**A**) Pre and 24 hr post drug basal OCR measurements of heart microtissues as % control n = 5, Stdev shown (Cyclophosphamide 200 uM, sorafenib 50 uM). (**B**) Pre- and 24 hr post drug basal PPR measurements of heart microtissues as % control n = 5, Stdev shown. Cyclophosphamide 200 uM, sorafenib 50 uM (**C**) Pre and 24 hr post drug basal OCR measurements of heart microtissues as % control n = 5, Stdev shown (2-DG 60 mM, BDM 30 mM). (**D**) Pre- and 24 hr post drug basal PPR measurements of heart microtissues as % control n = 5, Stdev shown (2-DG 60 mM, BDM 30 mM). (**E**) Pre and 24 hr post drug basal OCR measurements of heart microtissues as % control n = 5, Stdev shown. Zoledronic acid 1 mM, Oligomycin 1 uM, Rot/Ant A 2 uM (**F**) Pre and 24 hr post drug basal PPR measurements of heart microtissues as % control n = 5, Stdev shown (Zoledronic acid 1 mM, Oligomycin 1 uM, Rot/Ant A 2 uM).

To identify whether organ toxicity could be observed through changes in metabolic profile, we investigated the effects of cardiotoxins (sorafenib, cyclophosphamide and 2,3-Butane-dione monoxime (BDM)), nephrotoxins (zoledronic acid)^41^ and metabolic toxins (oligomycin, 2-deoxyglucose(2-DG) or rotenone and antimycin A combined), on organ microtissues. Heart and kidney microtissues were exposed to drugs for 24 hours, with pre- and post-treatment metabolic measurements- a pre-treatment basal metabolic profile, followed by a wash with media, and 24 hr. incubation in the presence of a drug, then a post-treatment profile. Figure 6A and B show that OCR and PPR are significantly reduced post-drug exposure with sorafenib, suggesting cardiotoxicity. Sorafenib is known to induce apoptosis in myocytes,, and this can induce destruction of electron transport chain, resulting in a decrease in both glycolysis and oxidative phosphorylation. Cyclophosphamide also has a significant effect on OCR, whereas PPR appears unaffected. However, PPR appearing the same as the control may be glycolytic burst before cell death. Heart microtissues treated with BDM, a known inhibitor of cardiac muscle contraction, became metabolically inhibited (Fig. 6C and D). Kidney microtissues exhibited reduced PPR and OCR in the presence of metabolic inhibitors oligomycin and rotenone/antimycin a (Fig. 6E and F) but zoledronic acid, a known nephrotoxin, although trended towards negatively effecting metabolism it was not significant. These data suggest drugs, which specifically damage the organ tissue or mechanism of function, could show metabolic defects through this type of assay before *in vivo* experiments or clinical trials. The increased knowledge of toxicity at an early stage could lead to optimization or alteration of compounds to limit this toxicity and further improve the likelihood of success in the clinic.

## Discussion

Utilizing this novel method, we demonstrate the ability to metabolically profile and observe subtle or substantial differences in the metabolic readout of 3D cell culture models, tumor and organ microtissues, and metabolic perturbations upon exposure to various drugs. Comparing the metabolic profile of 2D vs. 3D models, we observe differences in response to mitochondrial stress test drugs suggesting we should consider 3D models as a gold standard within research and pharmaceutical industries and may explain why some drugs fail in an *in vivo* model.

The 3D microtissues exhibited a delayed response to oligomycin which did not appear to be size mediated, as a substitution for a similar drug with a lower MW had the same delayed effect on OCR. We identify differences in metabolic phenotype when treating 2D and 3D cultures with common chemotherapeutics, such as 5-FU. 2D models are more sensitive than 3D, and often we observe the metabolic perturbation before viability decreases within these models.

We saw what appear to be protective rings of dead or dying cells around the spheroids, while the core remains intact even after prolonged exposure to a drug. Although this could not be rescued *in vitro*, it may give clues as to why, within *in vivo* models, drugs that have extremely high effectiveness in monolayer could be failing *in vivo*, as residual surviving cells remain. By profiling, we observe the densely packed nature of the spheroids is not the only protection used upon drug exposure, as we saw no improvement in the effect of therapy when using a microtubule-targeted drug that increased the penetrability of the spheroid. The different drug response demonstrates there are differences in the metabolism between 2D and 3D cell models and hence the effect of drugs on these. We attribute these differences to many reasons including altered cell: cell contact^42^, regional genotypic and phenotypic changes^30^ and diffusion gradients.

The ability to profile complex tissues such as organs and tumors can be revolutionary in delineating disease state, general metabolic health and the effect of pharmacological agents or microenvironmental pressures. We, coupled with physiological understanding, observe differences in microtissue metabolism and attribute this to cellular make-up. We have exhibited greater variance within the heart microtissues and would anticipate that separation of kidney cortex, and medulla microtissues would also have a more distinct metabolic phenotype. Greater understanding enables our ability to comprehend metabolic cross-talk which is becoming increasingly apparent in biological research including cancer, for example, stromal fibroblasts and tumor cells.

High-throughput metabolic analysis is more common practice and used to analyze many different diseases such as Alzheimer’s^43^, Friedrich’s Ataxia^44^ and cancer^23^. We showed with the production of our tool that metabolic flux analysis instruments can be redesigned and repurposed to metabolically profile 3D structures in a high-throughput manner with minimal variance and difficulty.

The number of products available to enable the production of 3D spheroid models and the utilization of these products has proven that it is a necessity, not an option, that we include 3D models in our studies^16^. Mathematical modeling^45^ and *in vitro* studies^28^ have shown that a 3D model recapitulates the microenvironmental, regional, gradient and cellular interactions better than a 2D model and much more akin to that observed in the tumor microenvironment, albeit an avascular tumor.

This robust assay combines 3D morphology with metabolic profiling to give reproducible and valuable information of cellular metabolic health, drug effects and organ metabolism and toxicity. Weinberg introduced metabolism as a key hallmark of cancer^46^, and yet the ability to profile it in the lab tends to be limited to 2 dimensions. Therefore, metabolic profiling of complex tissues can be utilized for numerous clinical and research aspects across multiple disease types and as proven, with comparable normal tissue analysis. Understanding the importance of 3D structure has been lost in the milieu of science but by regaining this understanding metabolic profiling of complex tissues can be utilized for multiple different aspects of both research, medicine and multiple disease types.

## Materials and Methods

### Spheroid Production

HCT116 (colon cancer) or A549 (NSCLC) cells were cultured in DMEM F12 or RPMI1640, respectively, supplemented with 5% FBS and 1% PenStrep. Cells were rinsed in PBS once confluent and detached with 1 ml of 0.05% trypsin and placed in a 37 °C incubator until detached. Once detached, 3 mL of normal growth media was added and cells were centrifuged at 900 rpm for 3 mins in a 15 mL falcon tube. Cell pellet was re-suspended in 5 mL of complete growth media and cells counted on an Invitrogen Countess following manufacturer’s protocol.

Spheroids were formed in Perfecta3D Hanging Drop 96-well Plates (3D Biomatrix). HCT116 cells were seeded at 5 K in 40 µL per handing drop in complete growth media and A549 cells at 5.5 K in 40 µL per hanging drop in complete growth media with 0.015% Phenol red free Matrigel matrix (Corning) using a multi-channel pipette. Upper and lower troughs of the hanging drop plate were filled with deionized water to reduce droplet evaporation. Plates were kept at 37 °C until spheroid formation, HCT116 spheroids form in 5–7 days and A549 spheroids form in 8–10 days. During that time water was replenished in the troughs and media in the wells (10 µL) as required. Once spheroids formed the upper plate containing spheroids was placed over a 96 well ultra-low attachment round -bottomed plate containing 150 µL of complete growth media in each well. Plates were centrifuged up to 300 rpm and allowed to stop with no brake. Plates containing spheroids were then placed at 37 °C for 2 days before any drug treatment or metabolic analysis.

### Microtissue Production

Generation of microtissues was performed on both organs and tumors from mice. The relative specimen was harvested from the mouse and placed directly in a falcon tube containing ice cold KHB buffer (12 mM D-Glucose, 2 mM L-Glutamine, 1 mM Sodium Pyruvate, 2 mM Sodium Citrate, 25 mM PIPES, 25 mM HEPES pH7.4) on ice. The specimen was then blotted dry and attached using cyanoacrylate to the base plate of a Leica VT1200 semiautomatic VT1200 vibrating blade microtome equipped with a sapphire knife (Ted Pella Cat# 121-18). Once fixed to the base plate, the specimen was covered in ice cold KHB buffer and ice packed into the surrounding reservoir. Settings used to cut slices were Thickness – 500 µm, Amplitude – 3 mm, Speed – 0.07 mm/s. Slices were then placed into a petri dish on ice containing KHB buffer. Microtissues were then generated by punching out sections of each slice with a 1 mm Hariss Uni-Core Punch and placing the resulting 1 mm diameter disks directly into a Seahorse XFe96 Spheroid Microplate containing either Seahorse media or growth media, depending on the experiment. Metabolic analysis and tests were then carried out using an Agilent Seahorse XFe96 Analyzer (Agilent Technologies).

### Spheroid and Microtissue Imaging

Spheroids and microtissues were imaged using Cytation 3 Imaging Reader (Biotek®) using Brightfield 4X and saved as “.tiff” files. These files were then imported into Definiens Developer Software and pixel count within the spheroids quantified using a designed program which detects spheroid border. Variance between spheroid sizes was not statistically significant when untreated.

### Metabolic Profiling

Metabolic profiling of microtissues and spheroids was undertaken using a Seahorse XFe96 Extracellular Flux Analyzer (Seahorse Bioscience) in XFe96 Spheroids Microplates.

Microtissues were placed directly into a microplate containing 160 µL per well of media (8.3 g/L DMEM Powder and 31 mM Na Chloride) supplemented with 12 mM D-Glucose, 2 mM L-Glutamine and 1 mM Na Pyruvate at pH 7.35. The plate was then placed in CO_2_ free incubator at 37 °C for 1 hour prior to metabolic analysis.

Spheroids were washed in complete growth media twice and then pipetted directly into XFe96 Spheroid Microplates containing 160uL of Seahorse XF Assay Medium supplemented with 6 mM D-Glucose and 1 mM L-Glutamine at pH 7.35 using the 8 well spheroid transfer system (Agilent Technologies) and a multi-channel pipette. Spheroids were then incubated in CO_2_ free incubator for 1 hr.

Spheroids and microtissues were centered into the micro-chamber inserts using a p200 pipette if not already naturally in the center of the well.

Basal oxygen consumption rates (OCR) and extracellular acidification rates/proton production rates (ECAR/PPR) were calculated using 2 mins mix and 4 mins measure cycles for up to 12 hours. Seahorse XF Cell Mito Stress Tests were carried out using sequential injections of Oligomycin (1 µM), FCCP (1 µM), and Rotenone plus Antimycin A (1 µM), with 6 cycles for Oligomycin as penetration within the spheroid or microtissue to allow an observed effect.

### Toxicity Studies

Mouse and heart microtissues were produced as described above and basal metabolic measurements taken in Seahorse Extracellular Flux Analyzer prior to drug exposure for 1 hour. Any microtissues not metabolically active were removed from the experiment before drug treatment. Media within the flux plate was replaced with Seahorse XF Assay Medium supplemented with 12 mM D-Glucose, 2 mM L-Glutamine and 1 mM Na Pyruvate for kidney microtissues or M-199 media supplemented with 5 mM creatine, 2 mM l-carnitine, 5 mM taurine, 12 mM glucose, 2 mM glutamine and 1 mM sodium pyruvate for the heart microtissues. Drugs were added to media prior to 24 hr incubation and post control basal metabolic measurements: BDM 30 mM, Sorafenib 50 µM, Zoledronic Acid 1 mM, Oligomycin 1 µM and Rotenone/Antimycin A (1 µM).Tissues were incubated in the corresponding media for 24 hours at 37 °C and then washed three times in Seahorse media containing 12 mM D-Glucose, 2 mM L-Glutamine and 1 mM Na Pyruvate. Microtissues were then placed back in Seahorse Extracellular Flux machine for post-drug exposure basal metabolic measurements for 10 hours.

Seahorse Wave software, Microsoft Excel 2010, and GraphPad were used to analyze metabolic data generated.

### Microtissue Pathology

Following metabolic analysis assay media was removed from the Spheroid Microplate leaving microtissue or spheroid undisturbed. 200 µL of fresh 10% formalin was added and left to fix for 24 hours at room temperature. Formalin was then removed and 200 µL of 70% isopropanol added until processing. Prior to processing the specimen was transferred into a 1.5 mL microcentrifuge tube. Specimen was then dehydrated in 70% ethanol 30 mins, 95% ethanol 30 mins and 100% ethanol 30 mins three times. Clearing was done with Xylene for 30 mins three times. Specimens were then embedded in paraffin wax after removal of xylene. Paraffin wax was melted at 60 °C, added to microcentrifuge tube and placed in a 60 °C water bath for 60 mins, repeated and then left in a water bath in the paraffin wax overnight. Once embedded and cooled, the microcentrifuge tube was cut to access spheroid and transferred into a disposable histology mold. Paraffin wax was then added to the mold attached to a histology cassette and place at 4 °C for 60 min. The embedded spheroid can then be sliced to generate slides for histological analysis.

Hematoxylin and Eosin staining was carried out on the embedded spheroids and microtissues.

### Data Analysis

Unpaired T- Test with Welch’s correction was performed using GraphPad Prism (version 6.00 for Windows, GraphPad Software, and La Jolla, California, USA). *p* values of 0.05 were deemed statistically significant. Values shown are mean ± SEM.

All experiments on live vertebrates were carried out in accordance with the guidelines and regulations set by University of South Florida (USF), Moffitt Cancer Center and the Institutional Animal Care and Use Committee (IACUC). The experimental protocols used were approved by USF and IACUC under protocol R IS00000736.

## Electronic supplementary material

Supplementary Data

## References

[1] Cori C, Cori G (1934). Carbohydrate metabolism. Annual Review of Biochemistry.

[2] Falkowska A (2015). Energy Metabolism of the Brain, Including the Cooperation between Astrocytes and Neurons, Especially in the Context of Glycogen Metabolism. International journal of molecular sciences.

[3] Argilés, J. M., Busquets, S. & López-Soriano, F. J. Metabolic interrelationships between liver and skeletal muscle in pathological states (2001). 10.1016/s0024-3205(01)01238-3 11531159

[4] Belanger M, Allaman I, Magistretti PJ (2011). Brain energy metabolism: focus on astrocyte-neuron metabolic cooperation. Cell metabolism.

[5] Salem AF (2012). Two-compartment tumor metabolism: autophagy in the tumor microenvironment and oxidative mitochondrial metabolism (OXPHOS) in cancer cells. Cell cycle.

[6] Perez-Escuredo J (2016). Lactate promotes glutamine uptake and metabolism in oxidative cancer cells. Cell cycle.

[7] Duggan SP (2006). Low pH induces co-ordinate regulation of gene expression in oesophageal cells. Carcinogenesis.

[8] Liou GY, Storz P (2010). Reactive oxygen species in cancer. Free radical research.

[9] Sun L (2015). cMyc-mediated activation of serine biosynthesis pathway is critical for cancer progression under nutrient deprivation conditions. Cell Res.

[10] Fraga A, Ribeiro R, Principe P, Lopes C, Medeiros R (2015). Hypoxia and Prostate Cancer Aggressiveness: A Tale With Many Endings. Clinical genitourinary cancer.

[11] Gillies RJ, Flowers CI, Drukteinis JS, Gatenby RA (2012). A unifying theory of carcinogenesis, and why targeted therapy doesn’t work. European Journal of Radiology.

[12] DiMasi JA, Reichert JM, Feldman L, Malins A (2013). Clinical approval success rates for investigational cancer drugs. Clinical pharmacology and therapeutics.

[13] Salanti A (2015). Targeting Human Cancer by a Glycosaminoglycan Binding Malaria Protein. Cancer cell.

[14] Weiswald LB, Bellet D, Dangles-Marie V (2015). Spherical cancer models in tumor biology. Neoplasia.

[15] Pampaloni, F., Reynaud, E. G. & Stelzer, E. H. K. The third dimension bridges the gap between cell culture and live tissue (2007). 10.1038/nrm2236 17684528

[16] Amann A (2014). Development of an innovative 3D cell culture system to study tumour–stroma interactions in non-small cell lung cancer cells. PloS one.

[17] Hess MW (2010). 3D versus 2D cell culture: implications for electron microscopy. Methods in cell biology.

[18] Anderson AR, Weaver AM, Cummings PT, Quaranta V (2006). Tumor morphology and phenotypic evolution driven by selective pressure from the microenvironment. Cell.

[19] Izuishi, K., Kato, K., Ogura, T., Kinoshita, T. & Esumi, H. Remarkable Tolerance of Tumor Cells to Nutrient Deprivation: Possible New Biochemical Target for Cancer Therapy (2001). 11085546

[20] Tredan O, Galmarini CM, Patel K, Tannock IF (2007). Drug resistance and the solid tumor microenvironment. Journal of the National Cancer Institute.

[21] Warburg, O. On the origin of cancer cells. *Science* **123** (1956). 10.1126/science.123.3191.309 13298683

[22] Bobrovnikova-Marjon E, Hurov JB (2014). Targeting metabolic changes in cancer: novel therapeutic approaches. Annual review of medicine.

[23] Simoes RV (2015). Metabolic plasticity of metastatic breast cancer cells: adaptation to changes in the microenvironment. Neoplasia.

[24] Cheng G (2014). Profiling and targeting of cellular bioenergetics: inhibition of pancreatic cancer cell proliferation. British journal of cancer.

[25] Bailey KM (2014). Mechanisms of buffer therapy resistance. Neoplasia.

[26] Pertega-Gomes, N. *et al*. A glycolytic phenotype is associated with prostate cancer progression and aggressiveness: A role for Monocarboxylate Transporters as metabolic targets for therapy. *The Journal of pathology*, 10.1002/path.4547 (2015). 10.1002/path.4547 PMC4528232 25875424

[27] Plas, D. R. & Thompson, C. B. Cell metabolism in the regulation of programmed cell death (2002). 10.1016/s1043-2760(01)00528-8 11854022

[28] Ferrick DA, Neilson A, Beeson C (2008). Advances in measuring cellular bioenergetics using extracellular flux. Drug discovery today.

[29] Tung YC (2011). High-throughput 3D spheroid culture and drug testing using a 384 hanging drop array. The Analyst.

[30] Shchepina, L. A. *et al*. Oligomycin, inhibitor of the F0 part of H + -ATP-synthase, suppresses the TNF-induced apoptosis. 10.1038/sj.onc (2002). 10.1038/sj.onc.1206053 12444550

[31] Goranova TE, Ohue M, Shimoharu Y, Kato K (2011). Dynamics of cancer cell subpopulations in primary and metastatic colorectal tumors. Clinical & experimental metastasis.

[32] Qian CN, Huang D, Wondergem B, Teh BT (2009). Complexity of tumor vasculature in clear cell renal cell carcinoma. Cancer.

[33] Zukin M (2013). Randomized phase III trial of single-agent pemetrexed versus carboplatin and pemetrexed in patients with advanced non-small-cell lung cancer and Eastern Cooperative Oncology Group performance status of 2. Journal of clinical oncology: official journal of the American Society of Clinical Oncology.

[34] Bambury RM (2015). The safety and efficacy of single-agent pemetrexed in platinum-resistant advanced urothelial carcinoma: a large single-institution experience. The oncologist.

[35] Douillard JY (2000). Irinotecan combined with fluorouracil compared with fluorouracil alone as first-line treatment for metastatic colorectal cancer: a multicentre randomised trial. The Lancet.

[36] Mehta G, Hsiao AY, Ingram M, Luker GD, Takayama S (2012). Opportunities and challenges for use of tumor spheroids as models to test drug delivery and efficacy. Journal of controlled release: official journal of the Controlled Release Society.

[37] Sui, H., Fan, Z.-Z. & Li, Q. Signal Transduction Pathways and Transcriptional Mechanisms of ABCB1/Pgp-mediated Multiple Drug Resistance in Human Cancer Cells (2012). 10.1177/147323001204000204 22613403

[38] King AE, Ackley MA, Cass CE, Young JD, Baldwin SA (2006). Nucleoside transporters: from scavengers to novel therapeutic targets. Trends in pharmacological sciences.

[39] Jia JB (2015). Chemotherapy-related complications in the kidneys and collecting system: an imaging perspective. Insights into imaging.

[40] Hammer KP, Hohendanner F, Blatter LA, Pieske BM, Heinzel FR (2014). Variations in local calcium signaling in adjacent cardiac myocytes of the intact mouse heart detected with two-dimensional confocal microscopy. Frontiers in physiology.

[41] Stopeck AT (2010). Denosumab compared with zoledronic acid for the treatment of bone metastases in patients with advanced breast cancer: a randomized, double-blind study. Journal of clinical oncology: official journal of the American Society of Clinical Oncology.

[42] Ichikawa Y (2014). Cardiotoxicity of doxorubicin is mediated through mitochondrial iron accumulation. The Journal of clinical investigation.

[43] Holscher C (2013). Age-Dependent Modulation of Synaptic Plasticity and Insulin Mimetic Effect of Lipoic Acid on a Mouse Model of Alzheimer’s Disease. PloS one.

[44] Richardson TE, Yu AE, Wen Y, Yang SH, Simpkins JW (2012). Estrogen prevents oxidative damage to the mitochondria in Friedreich’s ataxia skin fibroblasts. PloS one.

[45] Jiang Y, Pjesivac-Grbovic J, Cantrell C, Freyer JP (2005). A multiscale model for avascular tumor growth. Biophysical journal.

[46] Hanahan D, Weinberg RA (2011). Hallmarks of cancer: the next generation. Cell.

