# Supplementary material for: Metabolic Profiling of healthy and cancerous tissues in 2D and 3D
Source: Sci Rep. 2017 Nov 10;7:15285. doi: 10.1038/s41598-017-15325-5 (PMC5681543; doi:10.1038/s41598-017-15325-5)
Supplement: Supplementary file 1 — Supplementary Data [file 41598_2017_15325_MOESM1_ESM.pdf]

Title: **Metabolic Profiling of healthy and cancerous tissues in 2D and 3D**

Authors: Shonagh Russell[1,4], Jonathan Wojtkowiak[3], Andy Neilson[2], Robert J. Gillies[1]
Affiliations:
[1]Department of Cancer Imaging and Metabolism, H Lee Moffitt Cancer Centre and Research Institute, Tampa, FL, USA.
[2]Agilent Technologies (Seahorse Bioscience), 5301 Stevens Creek Blvd., Santa Clara, CA, 95051, USA.
[3]Genentech Inc., 15 Pemberton Rd, Wayland, MA, 01778, USA.
[4]University of South Florida, Tampa, FL, USA.
Corresponding Author:
Robert J. Gillies, Department of Imaging and Metabolism, Moffitt Cancer Centre, 12902 Magnolia Drive, Tampa, FL, USA, 33612. Email: Robert.gillies@moffitt.org

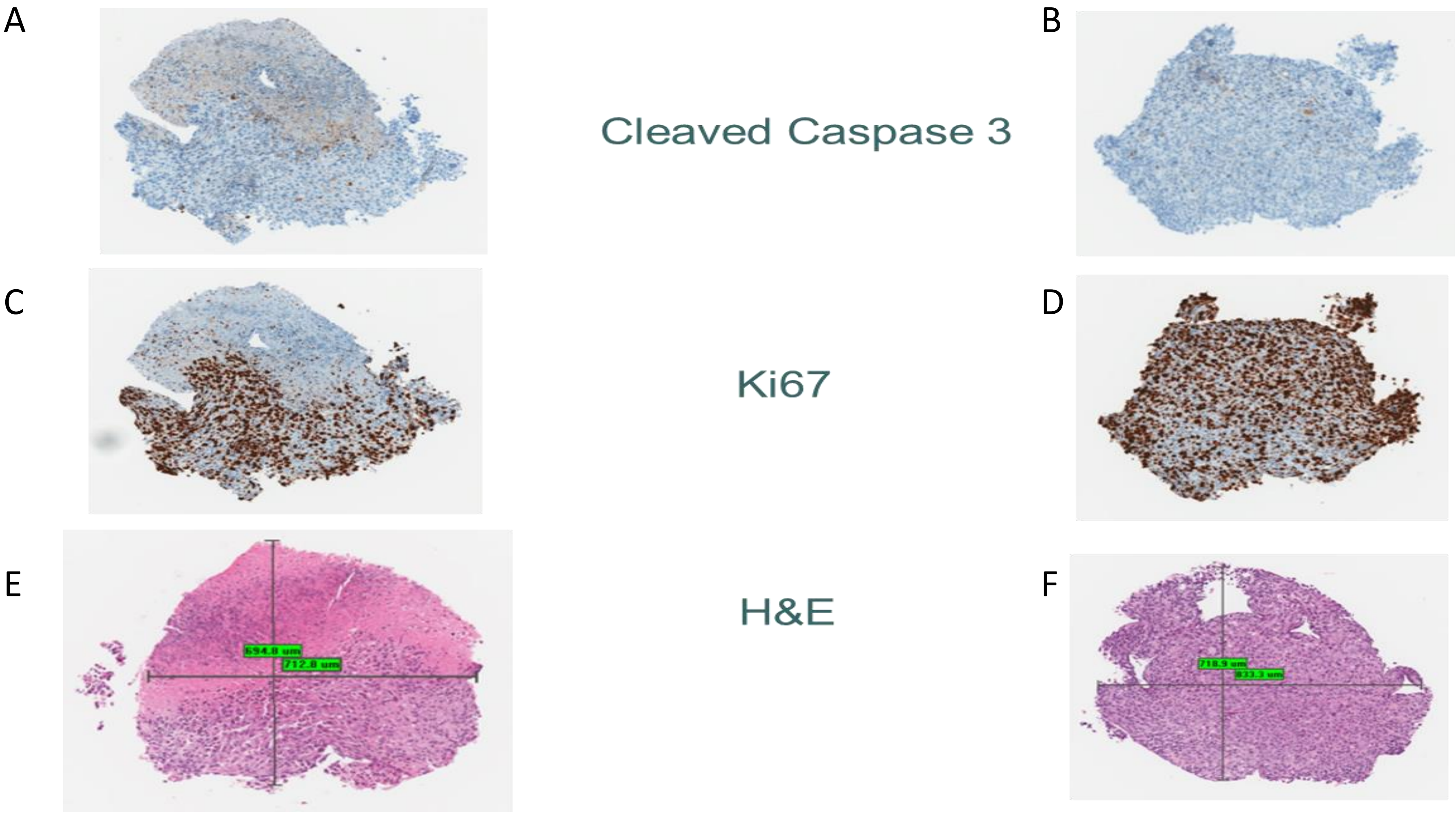

A
B
Cleaved Caspase 3
C
D
Ki67
E
F
H&E
694.8 um
712.8 um
718.9 um
833.3 um

Supplemental Figure 1: A-F: Immunohistochemistry staining for Cleaved Caspase 3, Ki67 and hematoxylin and eosin of 2 individual microtissues from HCT116 mouse xenograft tumors.

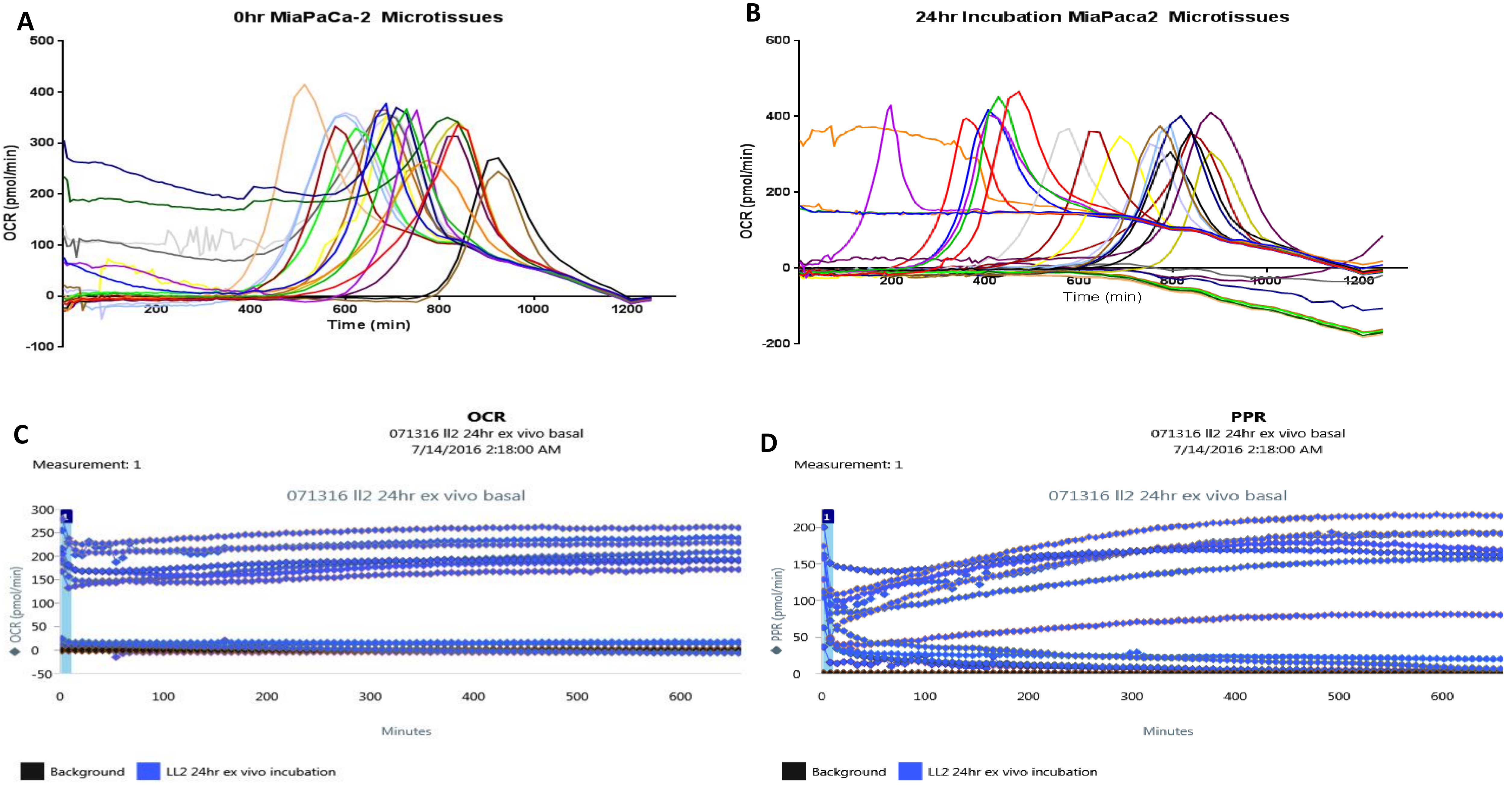

A
0hr MiaPaCa-2 Microtissues
OCR (pmol/min)
Time (min)
B
24hr Incubation MiaPaca2 Microtissues
OCR (pmol/min)
Time (min)
C
OCR
071316 ll2 24hr ex vivo basal
7/14/2016 2:18:00 AM
Measurement: 1
071316 ll2 24hr ex vivo basal
OCR (pmol/min)
Minutes
Background
LL2 24hr ex vivo incubation
D
PPR
071316 ll2 24hr ex vivo basal
7/14/2016 2:18:00 AM
Measurement: 1
071316 ll2 24hr ex vivo basal
PPR (pmol/min)
Minutes
Background
LL2 24hr ex vivo incubation

Supplementary Figure 2: Longevity studies of microtissues. A: 0hr *ex vivo* basal OCR measurements of MiaPaCa-2 microtissues. B: Post 24hr *ex vivo* incubation OCR rate of MiaPaCa-2 microtissues. C: Lewis lung cancer(LL2) microtissues post 24hr *ex vivo* incubation, basal OCR. D: Lewis lung cancer(LL2) microtissues post 24hr *ex vivo* incubation, basal PPR.

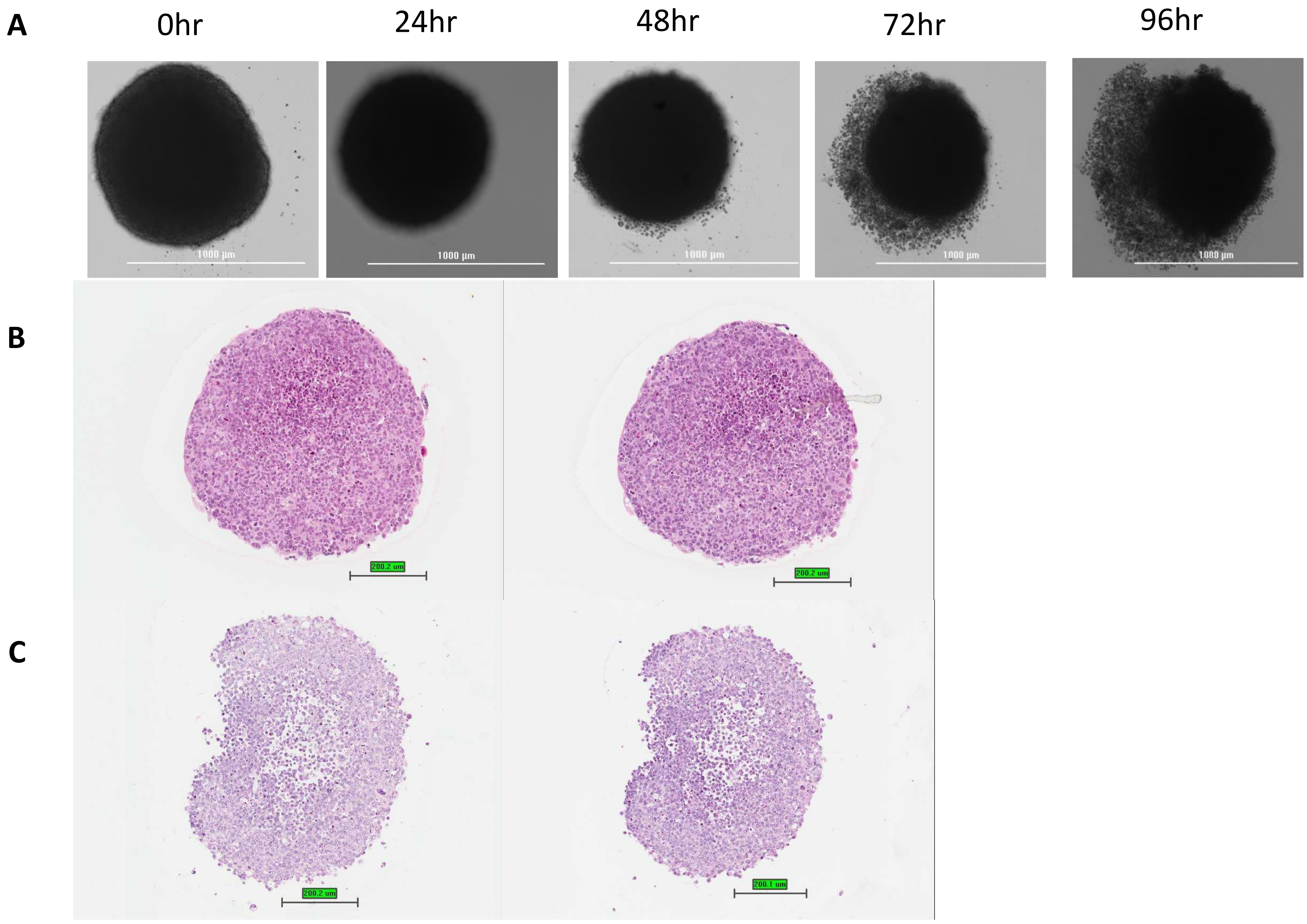
A
0hr
24hr
48hr
72hr
96hr
1000 µm
B
200.2 um
C
200.2 um
200.1 um

Supplementary Figure 3: **HCT116 Spheroids with 5FU Treatment**. A: Brightfield images of HCT116 spheroids treated with 2mM 5-FU for 96 hours, taken every 24 hours showing disruption of outer layer of cells. After removal of 5-FU treatment spheroids were unable to recover, cells disaggregated. N=6 per time point. Representative images shown. B: H & E staining of HCT116 spheroids untreated. C: H & E staining of HCT116 spheroids treated with 2mM 5Fu for 96hrs.

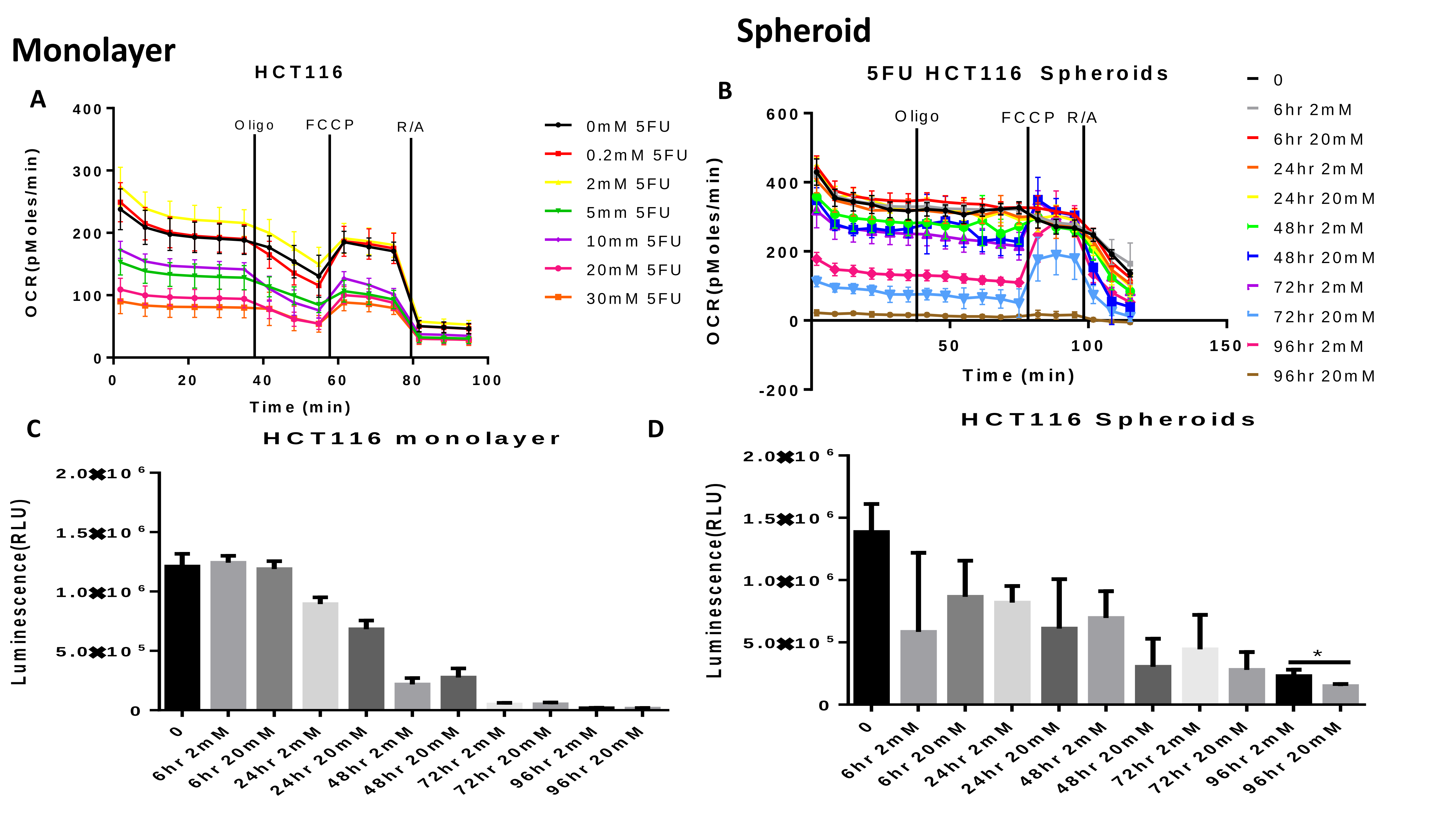
Monolayer
Spheroid
A
HCT116
OCR(pMoles/min)
Time (min)
Oligo
FCCP
R/A
0mM 5FU
0.2mM 5FU
2mM 5FU
5mm 5FU
10mm 5FU
20mM 5FU
30mM 5FU
B
5FU HCT116 Spheroids
OCR(pMoles/min)
Time (min)
Oligo
FCCP
R/A
0
6hr 2mM
6hr 20mM
24hr 2mM
24hr 20mM
48hr 2mM
48hr 20mM
72hr 2mM
72hr 20mM
96hr 2mM
96hr 20mM
C
HCT116 monolayer
Luminescence(RLU)
0
6hr 2mM
6hr 20mM
24hr 2mM
24hr 20mM
48hr 2mM
48hr 20mM
72hr 2mM
72hr 20mM
96hr 2mM
96hr 20mM
D
HCT116 Spheroids
Luminescence(RLU)
*
0
6hr 2mM
6hr 20mM
24hr 2mM
24hr 20mM
48hr 2mM
48hr 20mM
72hr 2mM
72hr 20mM
96hr 2mM
96hr 20mM

Supplementary Figure 4:**Effects of 5FU on 3D spheroid metabolic phenotype versus 2D monolayer in HCT116 colorectal cancer cell line.** A: Mitochondrial stress test on HCT116 monolayer treated with increasing 5FU concentrations, 0-30mM for 48hr. N=5 per group SD. B: Time course of 5FU treatment with 2mM or 20mM on HCT116 spheroids. Concentration decided from monolayer data. N=5 per group, SEM shown. C: Cell titer-Glo Luminescent Cell Viability ATP assay of HCT116 monolayer over 96hrs with 2 or 20mM 5FU treatment. D: Cell titer-Glo Luminescent Cell Viability ATP assay of HCT116 spheroid over 96hrs with 2 or 20mM 5FU treatment. N=5, SD shown.

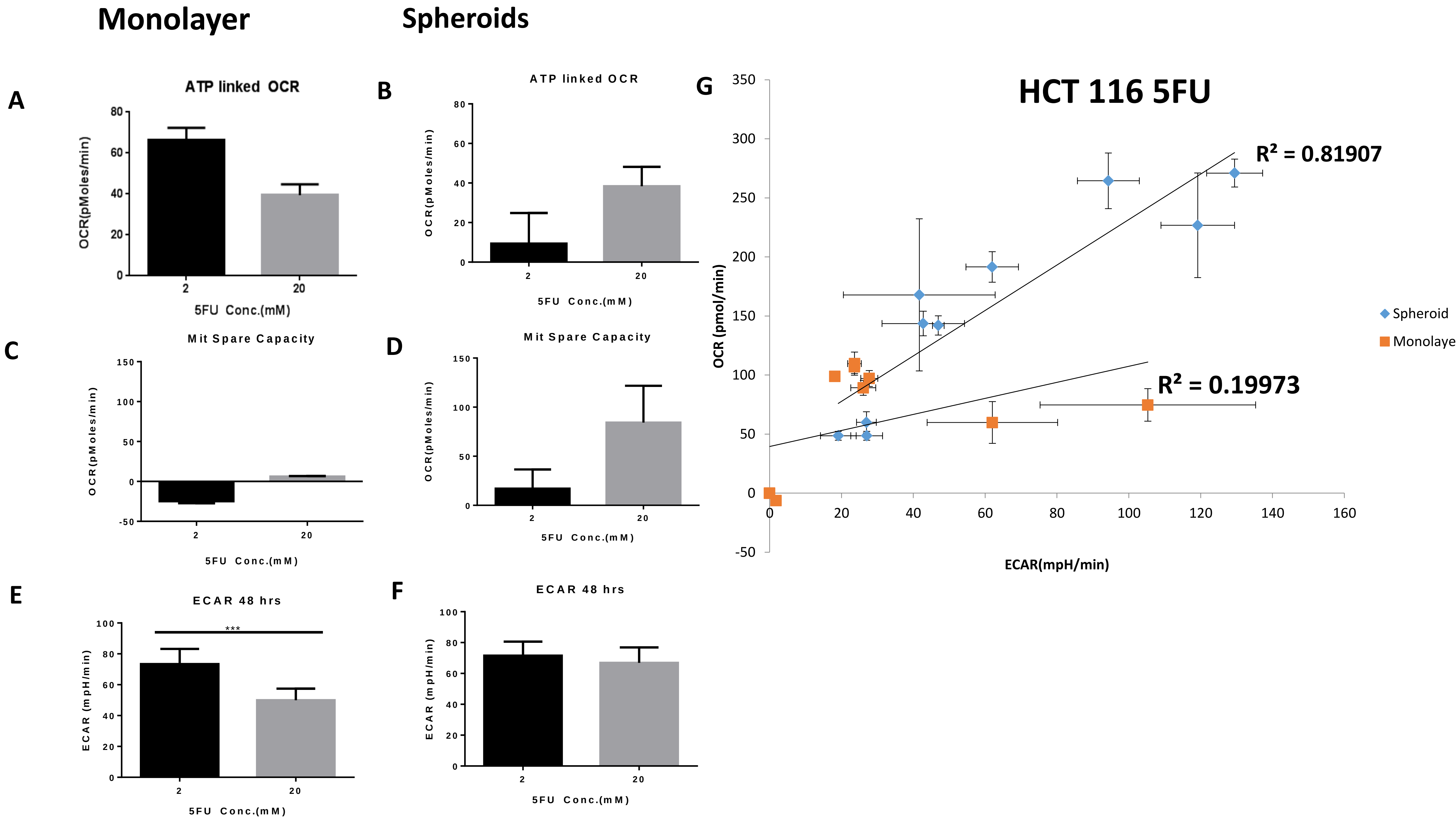

Monolayer
Spheroids
A
ATP linked OCR
OCR(pMoles/min)
5FU Conc.(mM)
B
ATP linked OCR
OCR(pMoles/min)
5FU Conc.(mM)
C
Mit Spare Capacity
OCR(pMoles/min)
5FU Conc.(mM)
D
Mit Spare Capacity
OCR(pMoles/min)
5FU Conc.(mM)
E
ECAR 48 hrs
***
ECAR (mpH/min)
5FU Conc.(mM)
F
ECAR 48 hrs
ECAR (mpH/min)
5FU Conc.(mM)
G
HCT 116 5FU
R² = 0.81907
R² = 0.19973
OCR (pmol/min)
ECAR(mpH/min)
Spheroid
Monolaye

Supplementary Figure 5: **HCT 116 monolayer and spheroid metabolic phenotype after 48hr 5FU Treatment.** A:HCT116 monolayer ATP linked OCR with 5FU treatment. B: HCT116 spheroid ATP linked OCR with 5FU treatment. ATP linked OCR determined from difference between basal OCR and decrease upon oligomycin addition C: HCT116 monolayer mitochondrial spare respiratory capacity with 5FU treatment. D:HCT116 spheroid mitochondrial spare respiratory capacity with 5FU treatment. E: Basal ECAR of HCT116 monolayer prior to stress test after 48 hrs 5FU treatment. F: Basal ECAR of HCT116 spheroid monolayer after 48 hrs 5FU treatment. N=5, SEM shown. ***- p<0.00. G: OCR: ECAR energy profile of HCT116 spheroid vs monolayer of response to 5FU treatment.

HCT-116 Rapamycin 24hr

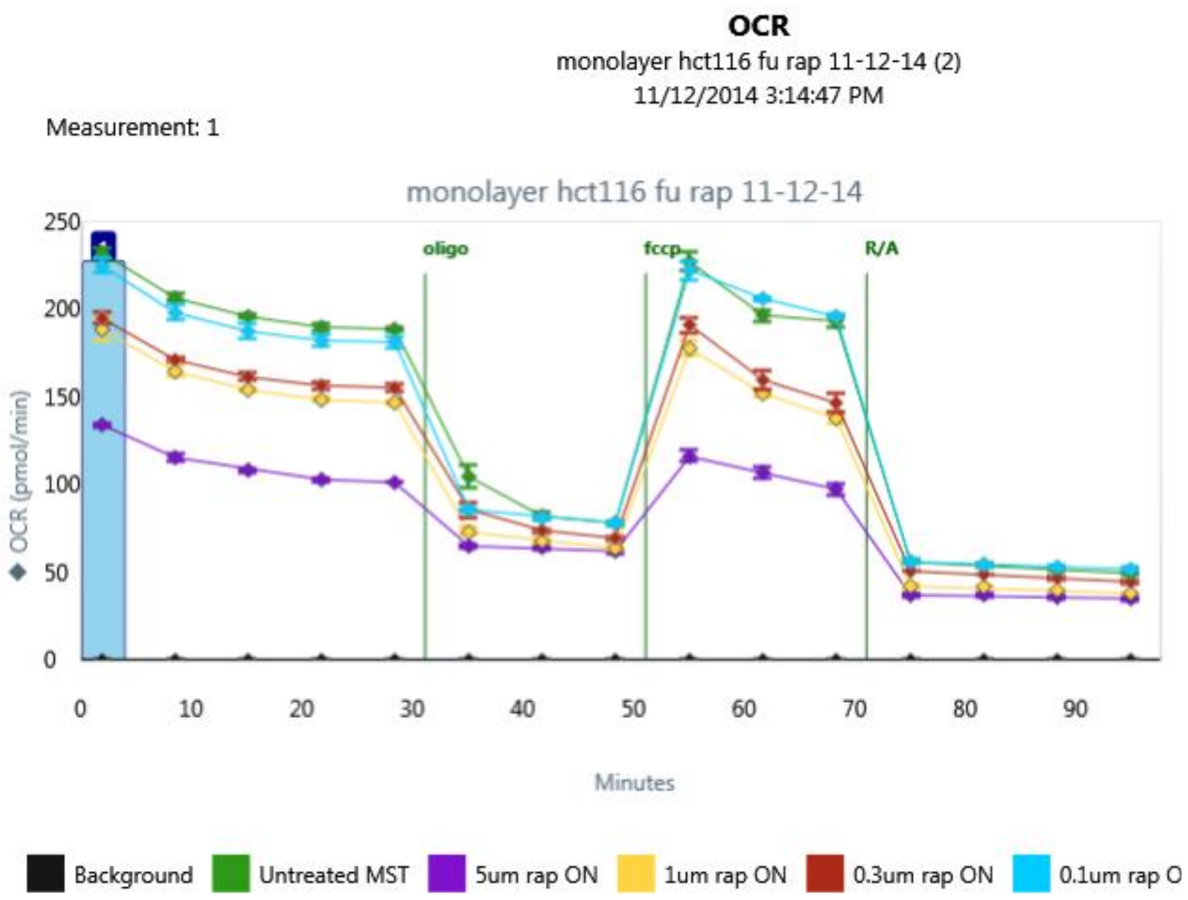

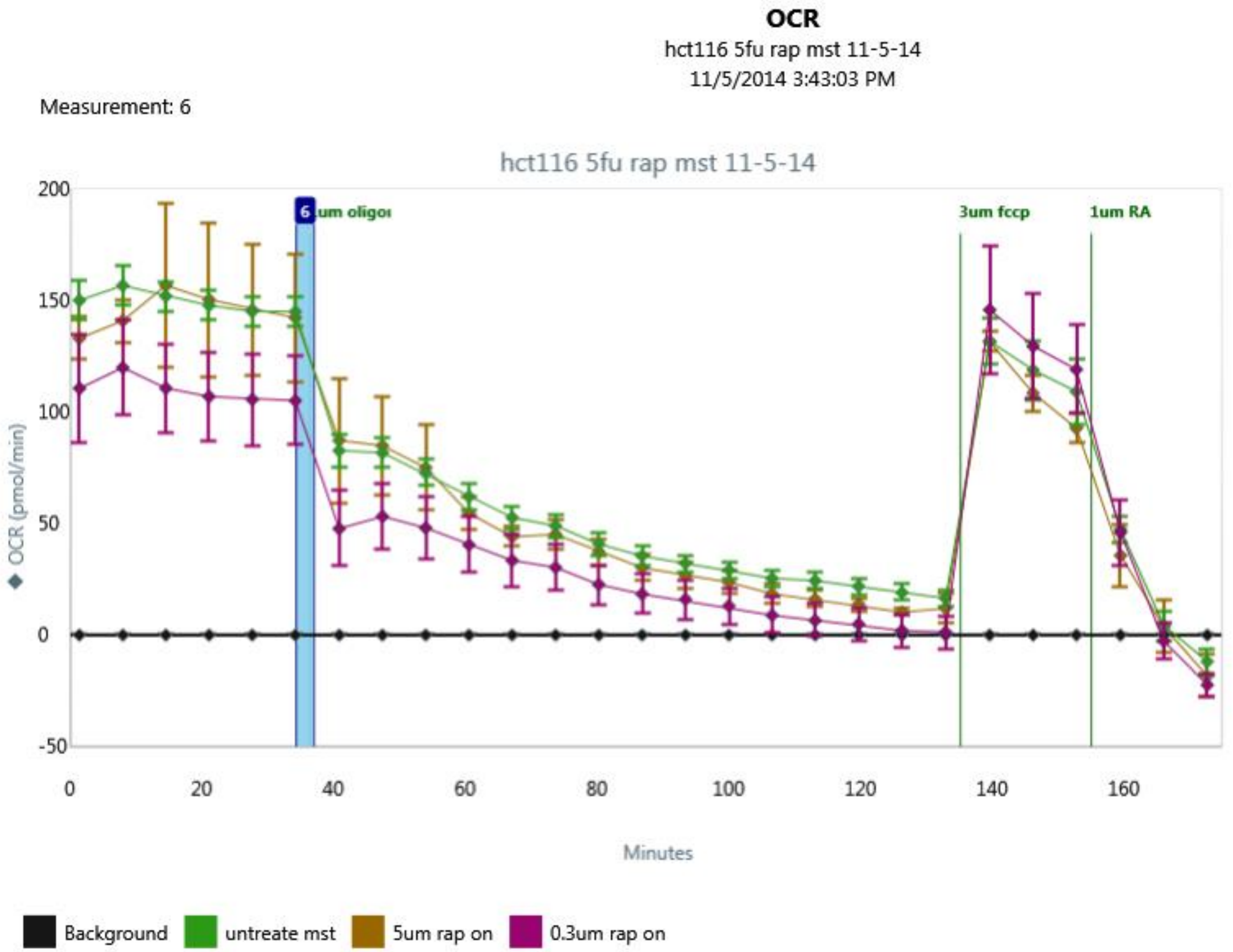

Supplementary Figure 6:**Effects of rapamycin on 3D spheroid metabolic phenotype versus 2D monolayer in HCT116 colorectal cancer cell line.** A: Mitochondrial stress test on HCT116 monolayer treated with increasing rapamycin concentrations, 0-5µM overnight. N=5 per group SD. B: Mitochondrial stress test on HCT116 spheroids treated with 5µM or 0.3µM of rapamycin overnight. N=5 per group, SEM shown.

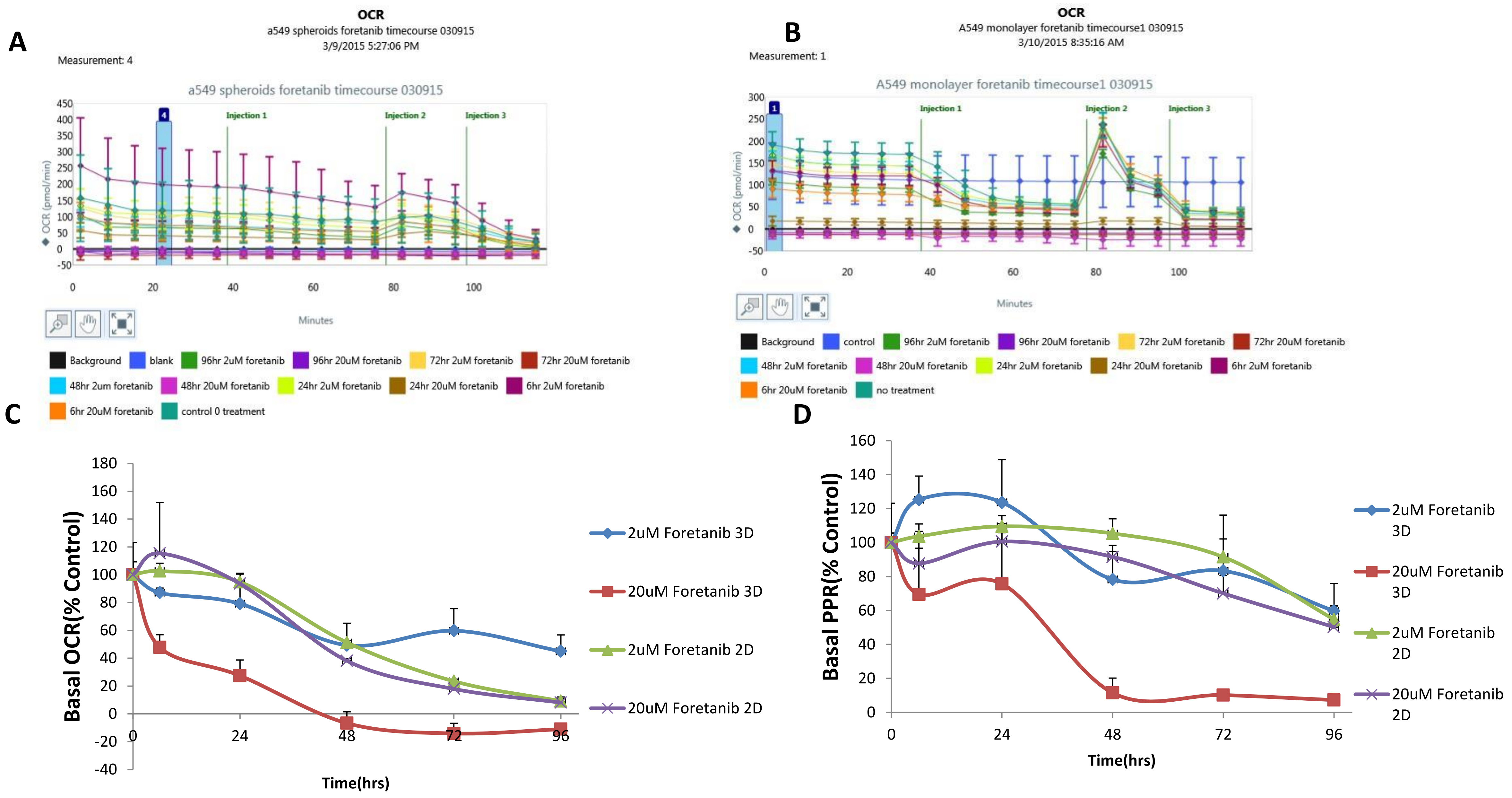
A
OCR
a549 spheroids foretanib timecourse 030915
3/9/2015 5:27:06 PM
Measurement: 4
a549 spheroids foretanib timecourse 030915
OCR (pmol/min)
Injection 1
Injection 2
Injection 3
Minutes
Background
blank
96hr 2uM foretanib
96hr 20uM foretanib
72hr 2uM foretanib
72hr 20uM foretanib
48hr 2um foretanib
48hr 20uM foretanib
24hr 2uM foretanib
24hr 20uM foretanib
6hr 2uM foretanib
6hr 20uM foretanib
control 0 treatment
B
OCR
A549 monolayer foretanib timecourse1 030915
3/10/2015 8:35:16 AM
Measurement: 1
A549 monolayer foretanib timecourse1 030915
OCR (pmol/min)
Injection 1
Injection 2
Injection 3
Minutes
Background
control
96hr 2uM foretanib
96hr 20uM foretanib
72hr 2uM foretanib
72hr 20uM foretanib
48hr 2uM foretanib
48hr 20uM foretanib
24hr 2uM foretanib
24hr 20uM foretanib
6hr 2uM foretanib
6hr 20uM foretanib
no treatment
C
Basal OCR(% Control)
Time(hrs)
2uM Foretanib 3D
20uM Foretanib 3D
2uM Foretanib 2D
20uM Foretanib 2D
D
Basal PPR(% Control)
Time(hrs)
2uM Foretanib 3D
20uM Foretanib 3D
2uM Foretanib 2D
20uM Foretanib 2D

Supplemental Figure 7: Effects of foretanib on 3D spheroid metabolic phenotype versus 2D monolayer in A549 human lung adenocarcinoma epithelial cell line. A: Mitochondrial stress test on A549 monolayer treated with 2uM or 20uM o foretanib over 96hrs. N=5 per group, SD shown. B: Mitochondrial stress test on A549 spheroids treated with 2uM or 20uM of foretanib over 96hrs. N=5 per group, SD shown. C: Basal OCR as % control (from measurement 6) at each time point with 2 or 20uM foretanib treatment, 2D vs 3D. D: Basal PPR as % control (from measurement 6) at each time point with 2 or 20uM foretanib treatment, 2D vs 3D.

A549

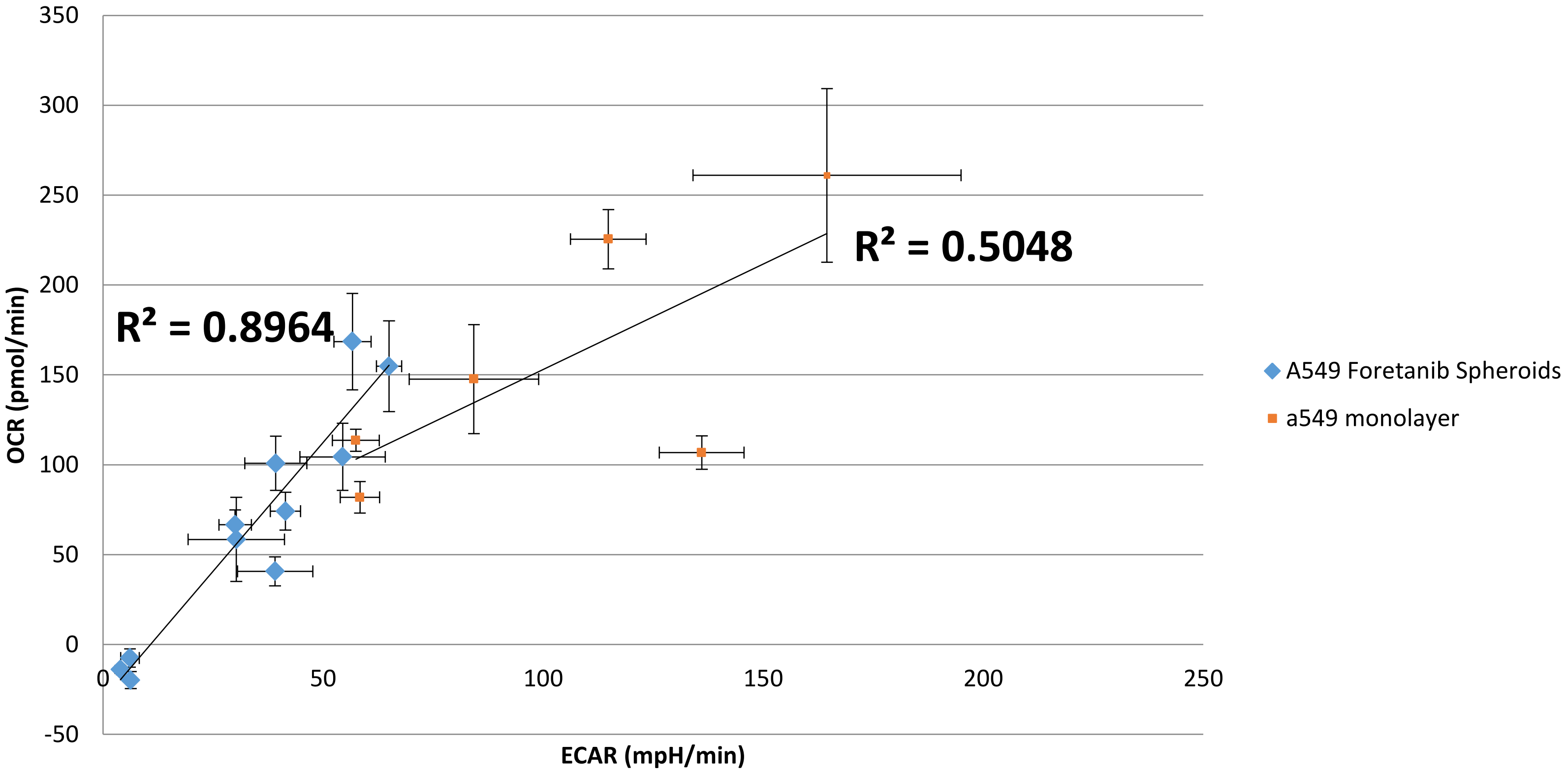

Foretanib is an ATP competitive inhibitor of VEGFR and HGFR. Involved in angiogensesis, migration, proliferation.

Supplemental Figure 7 (Cont.) E: OCR: ECAR energy profile of A549 spheroid vs monolayer in response to 20uM foretanib treatment over 96hrs. SD shown.

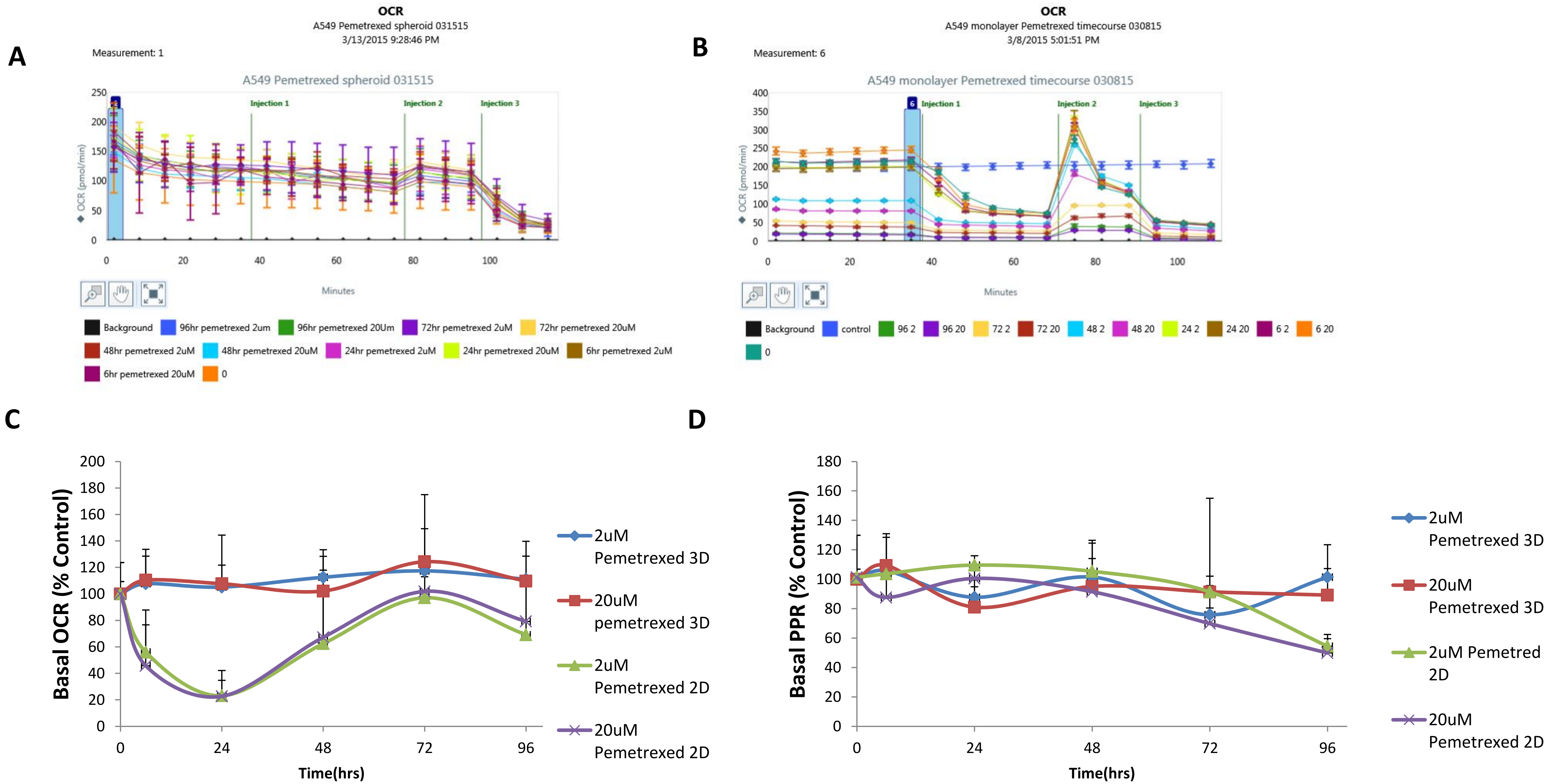
A
OCR
A549 Pemetrexed spheroid 031515
3/13/2015 9:28:46 PM
Measurement: 1
A549 Pemetrexed spheroid 031515
Injection 1
Injection 2
Injection 3
OCR (pmol/min)
Minutes
Background
96hr pemetrexed 2um
96hr pemetrexed 20Um
72hr pemetrexed 2uM
72hr pemetrexed 20uM
48hr pemetrexed 2uM
48hr pemetrexed 20uM
24hr pemetrexed 2uM
24hr pemetrexed 20uM
6hr pemetrexed 2uM
6hr pemetrexed 20uM
0
B
OCR
A549 monolayer Pemetrexed timecourse 030815
3/8/2015 5:01:51 PM
Measurement: 6
A549 monolayer Pemetrexed timecourse 030815
Injection 1
Injection 2
Injection 3
OCR (pmol/min)
Minutes
Background
control
96 2
96 20
72 2
72 20
48 2
48 20
24 2
24 20
6 2
6 20
0
C
Basal OCR (% Control)
Time(hrs)
2uM Pemetrexed 3D
20uM pemetrexed 3D
2uM Pemetrexed 2D
20uM Pemetrexed 2D
D
Basal PPR (% Control)
Time(hrs)
2uM Pemetrexed 3D
20uM Pemetrexed 3D
2uM Pemetred 2D
20uM Pemetrexed 2D

Supplemental Figure 8: Effects of pemetrexed on 3D spheroid metabolic phenotype versus 2D monolayer in A549 human lung adenocarcinoma epithelial cell line. A: Mitochondrial stress test on A549 monolayer treated with 2uM or 20uM pemetrexed over 96hrs. N=5 per group, SD shown. B: Mitochondrial stress test on A549 spheroids treated with 2uM or 20uM pemetrexed over 96hrs. N=5 per group, SD shown. C: Basal OCR as % control (from measurement 6) at each time point with 2 or 20uM pemetrexed treatment, 2D vs 3D. D: Basal PPR as % control (from measurement 6) at each time point with 2 or 20uM pemetrexed treatment, 2D vs 3D.

A

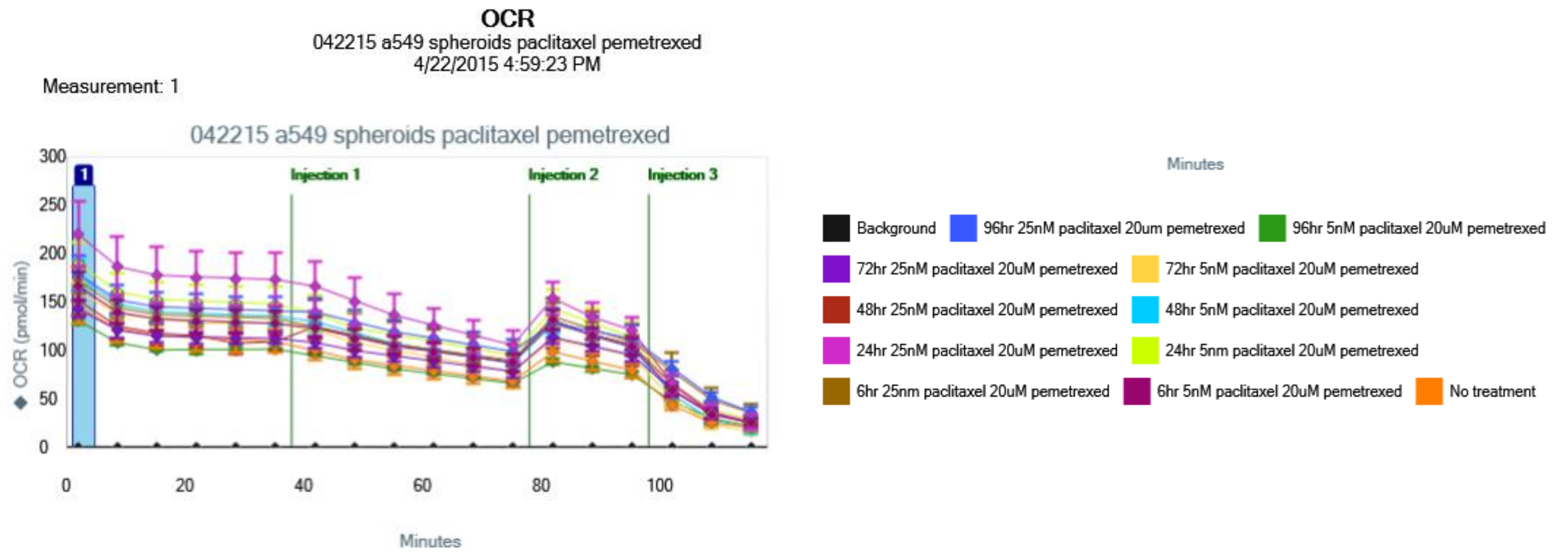
OCR
042215 a549 spheroids paclitaxel pemetrexed
4/22/2015 4:59:23 PM
Measurement: 1
042215 a549 spheroids paclitaxel pemetrexed
Injection 1
Injection 2
Injection 3
OCR (pmol/min)
Minutes
Background
96hr 25nM paclitaxel 20um pemetrexed
96hr 5nM paclitaxel 20uM pemetrexed
72hr 25nM paclitaxel 20uM pemetrexed
72hr 5nM paclitaxel 20uM pemetrexed
48hr 25nM paclitaxel 20uM pemetrexed
48hr 5nM paclitaxel 20uM pemetrexed
24hr 25nM paclitaxel 20uM pemetrexed
24hr 5nm paclitaxel 20uM pemetrexed
6hr 25nm paclitaxel 20uM pemetrexed
6hr 5nM paclitaxel 20uM pemetrexed
No treatment

B

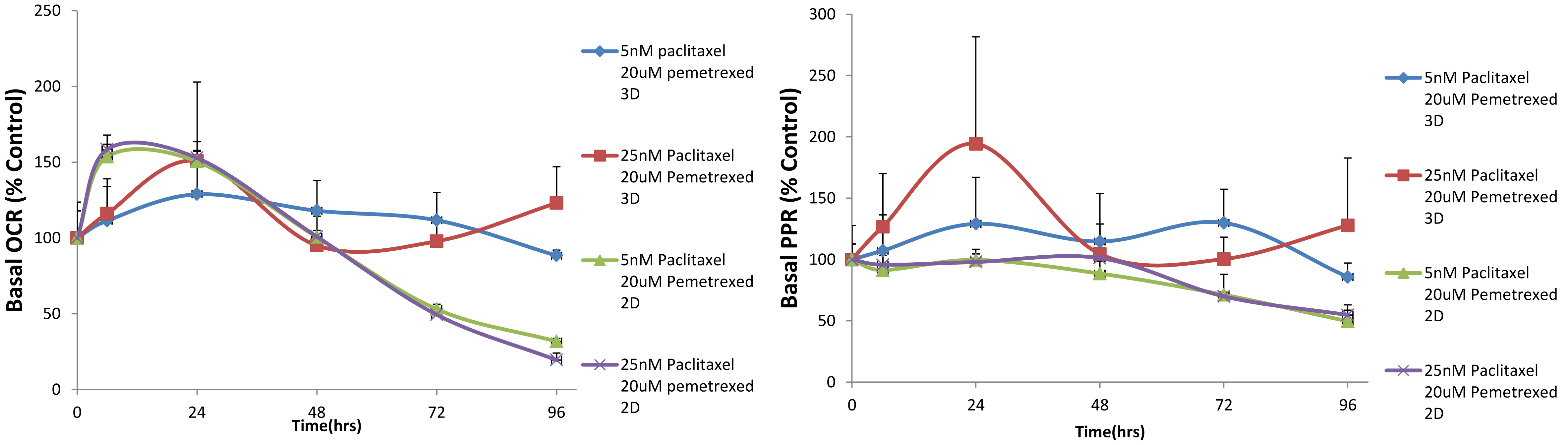
Basal OCR (% Control)
Time(hrs)
5nM paclitaxel 20uM pemetrexed 3D
25nM Paclitaxel 20uM Pemetrexed 3D
5nM Paclitaxel 20uM Pemetrexed 2D
25nM Paclitaxel 20uM pemetrexed 2D
Basal PPR (% Control)
Time(hrs)
5nM Paclitaxel 20uM Pemetrexed 3D
25nM Paclitaxel 20uM Pemetrexed 3D
5nM Paclitaxel 20uM Pemetrexed 2D
25nM Paclitaxel 20uM Pemetrexed 2D

Supplementary Figure 9: **Effects of dual treatment with paclitaxel and pemetrexed on spheroid metabolism**. A: Mitochondrial stress test of HCT116 spheroids treated in combination with 5 or 25nM paclitaxel and 20uM pemetrexed. B: Basal OCR, % control of monolayer and spheroid HCT116 with 5 or 25nM paclitaxel plus 20uM pemetrexed treatment for 48hr. C: Basal PPR, % control of monolayer and spheroid HCT116 with 5 or 25nM paclitaxel plus 20uM pemetrexed treatment for 48hr.

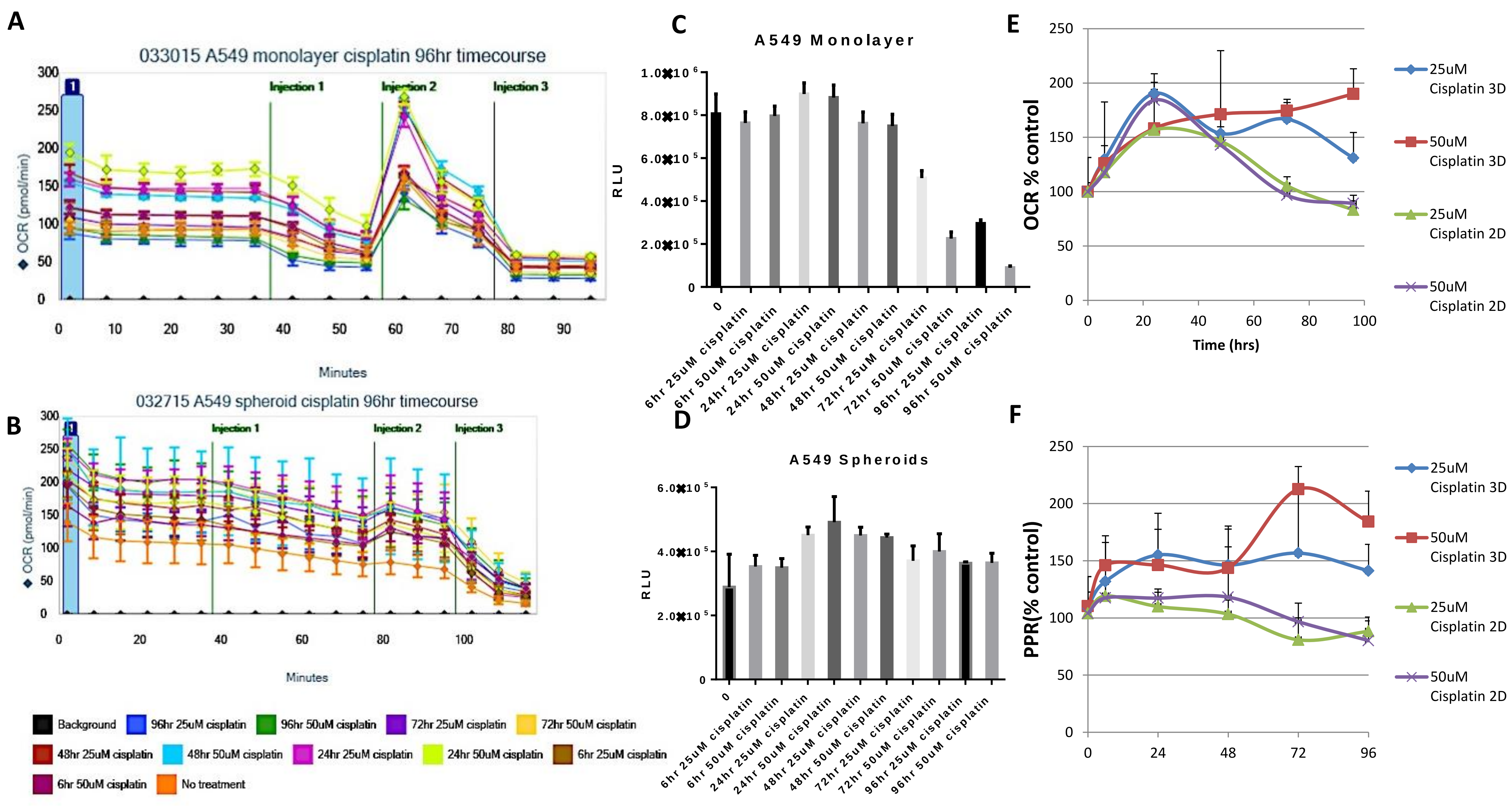
A
033015 A549 monolayer cisplatin 96hr timecourse
Injection 1
Injection 2
Injection 3
OCR (pmol/min)
Minutes
B
032715 A549 spheroid cisplatin 96hr timecourse
Injection 1
Injection 2
Injection 3
OCR (pmol/min)
Minutes
Background
96hr 25uM cisplatin
96hr 50uM cisplatin
72hr 25uM cisplatin
72hr 50uM cisplatin
48hr 25uM cisplatin
48hr 50uM cisplatin
24hr 25uM cisplatin
24hr 50uM cisplatin
6hr 25uM cisplatin
6hr 50uM cisplatin
No treatment
C
A549 Monolayer
RLU
0
6hr 25uM cisplatin
6hr 50uM cisplatin
24hr 25uM cisplatin
24hr 50uM cisplatin
48hr 25uM cisplatin
48hr 50uM cisplatin
72hr 25uM cisplatin
72hr 50uM cisplatin
96hr 25uM cisplatin
96hr 50uM cisplatin
D
A549 Spheroids
RLU
E
OCR % control
Time (hrs)
25uM Cisplatin 3D
50uM Cisplatin 3D
25uM Cisplatin 2D
50uM Cisplatin 2D
F
PPR(% control)
25uM Cisplatin 3D
50uM Cisplatin 3D
25uM Cisplatin 2D
50uM Cisplatin 2D

Supplemental Figure 10: Effects of cisplatin on 3D spheroid metabolic phenotype versus 2D monolayer in A549 human lung adenocarcinoma epithelial cell line. A: Mitochondrial stress test on A549 monolayer treated with 25uM or 50uM cisplatin over 96hrs. N=5 per group, SD shown. B: Mitochondrial stress test on A549 spheroids treated with 25uM or 50uM cisplatin over 96hrs. N=5 per group, SD shown. C: Cell titer-Glo Luminescent Cell Viability ATP assay of A549 monolayer over 96hrs with 25 or 50uM cisplatin treatment. B: Cell titer-Glo Luminescent Cell Viability ATP assay of A549 spheroid over 96hrs with 25 or 50uM cisplatin treatment. N=5, SD shown.E: Basal OCR as % control (from measurement 6) at each time point with 25 or 50uM cisplatin treatment, 2D vs 3D. F: Basal PPR as % control (from measurement 6) at each time point with 25 or 50uM cisplatin treatment, 2D vs 3D.

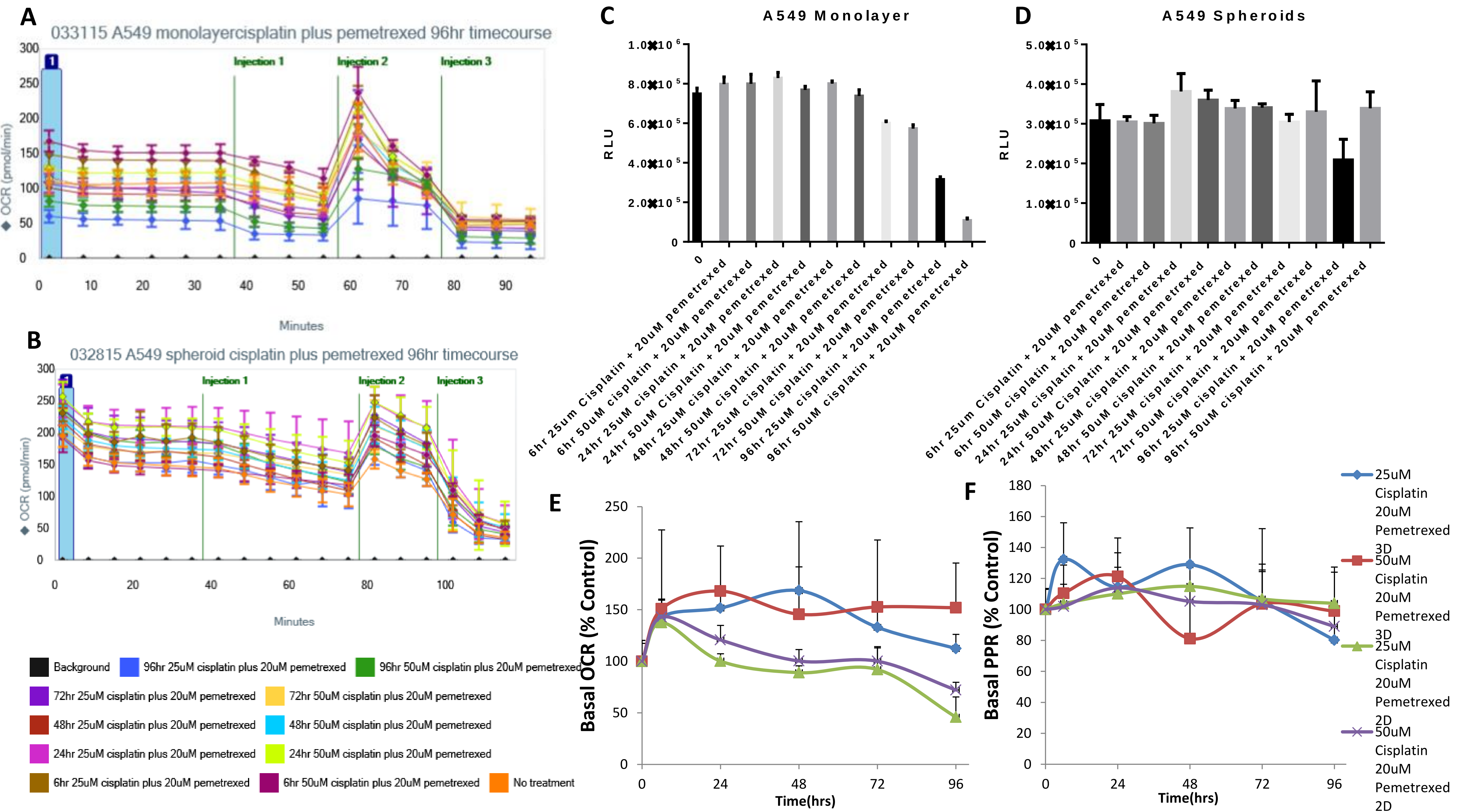
A
033115 A549 monolayercisplatin plus pemetrexed 96hr timecourse
Injection 1
Injection 2
Injection 3
OCR (pmol/min)
Minutes
B
032815 A549 spheroid cisplatin plus pemetrexed 96hr timecourse
Background
96hr 25uM cisplatin plus 20uM pemetrexed
96hr 50uM cisplatin plus 20uM pemetrexed
72hr 25uM cisplatin plus 20uM pemetrexed
72hr 50uM cisplatin plus 20uM pemetrexed
48hr 25uM cisplatin plus 20uM pemetrexed
48hr 50uM cisplatin plus 20uM pemetrexed
24hr 25uM cisplatin plus 20uM pemetrexed
24hr 50uM cisplatin plus 20uM pemetrexed
6hr 25uM cisplatin plus 20uM pemetrexed
6hr 50uM cisplatin plus 20uM pemetrexed
No treatment
C
A549 Monolayer
D
A549 Spheroids
RLU
6hr 25um Cisplatin + 20uM pemetrexed
6hr 50uM cisplatin + 20uM pemetrexed
24hr 25uM cisplatin + 20uM pemetrexed
24hr 50uM Cisplatin + 20uM pemetrexed
48hr 25uM cisplatin + 20uM pemetrexed
48hr 50uM cisplatin + 20uM pemetrexed
72hr 25uM cisplatin + 20uM pemetrexed
72hr 50uM cisplatin + 20uM pemetrexed
96hr 25uM cisplatin + 20uM pemetrexed
96hr 50uM cisplatin + 20uM pemetrexed
E
Basal OCR (% Control)
Time(hrs)
F
Basal PPR (% Control)
Time(hrs)
25uM Cisplatin 20uM Pemetrexed 3D
50uM Cisplatin 20uM Pemetrexed 3D
25uM Cisplatin 20uM Pemetrexed 2D
50uM Cisplatin 20uM Pemetrexed 2D

Supplemental Figure 11: Effects of cisplatin and pemetrexed on 3D spheroid metabolic phenotype versus 2D monolayer in A549 human lung adenocarcinoma epithelial cell line. A: Mitochondrial stress test on A549 monolayer treated with 25uM or 50uM cisplatin plus 20uM pemetrexed over 96hrs. N=5 per group, SD shown. B: Mitochondrial stress test on A549 spheroids treated with 25uM or 50uM cisplatin plus 20uM pemetrexed over 96hrs. N=5 per group, SD shown. C: Cell titer-Glo Luminescent Cell Viability ATP assay of A549 monolayer over 96hrs with 25 or 50uM cisplatin treatment plus 20uM pemetrexed . D: Cell titer-Glo Luminescent Cell Viability ATP assay of A549 spheroid over 96hrs with 25 or 50uM cisplatin treatment plus 20uM pemetrexed . N=5, SD shown. E: Basal OCR as % control (from measurement 6) at each time point with 25 or 50uM cisplatin treatment plus 20uM pemetrexed , 2D vs 3D. F: Basal PPR as % control (from measurement 6) at each time point with 25 or 50uM cisplatin treatment plus 20uM pemetrexed , 2D vs 3D.

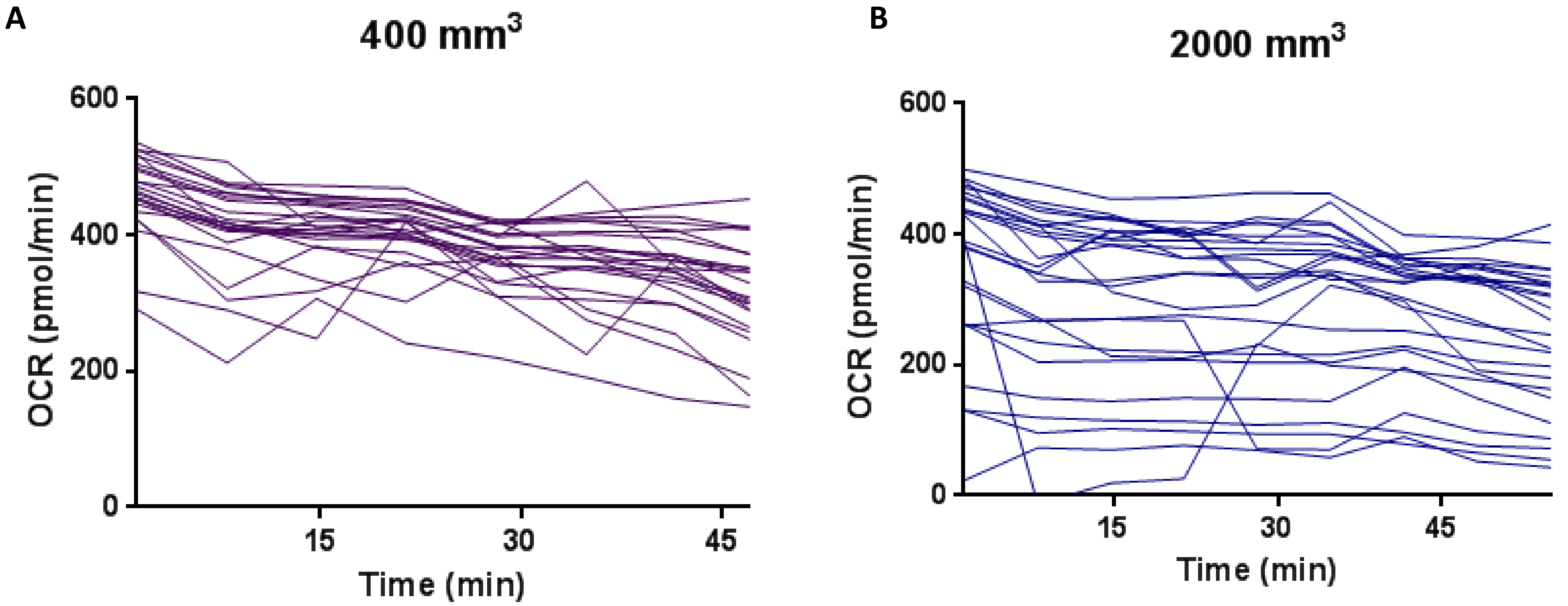
A
400 mm3
600
400
200
0
OCR (pmol/min)
15
30
45
Time (min)
B
2000 mm3
600
400
200
0
OCR (pmol/min)
15
30
45
Time (min)

Supplementary Figure 12: **Metabolic Heterogeneity of MiaPaCa-2 Tumor Microtissues** A: OCR of microtissues generated from a 400mm^3 tumor with Injection 1 (FCCP 1uM) and Injection 2 (Rotenone/Antimycin A 1uM) inhibitors. B: OCR of microtissues generated from a 2000mm^3 tumor.

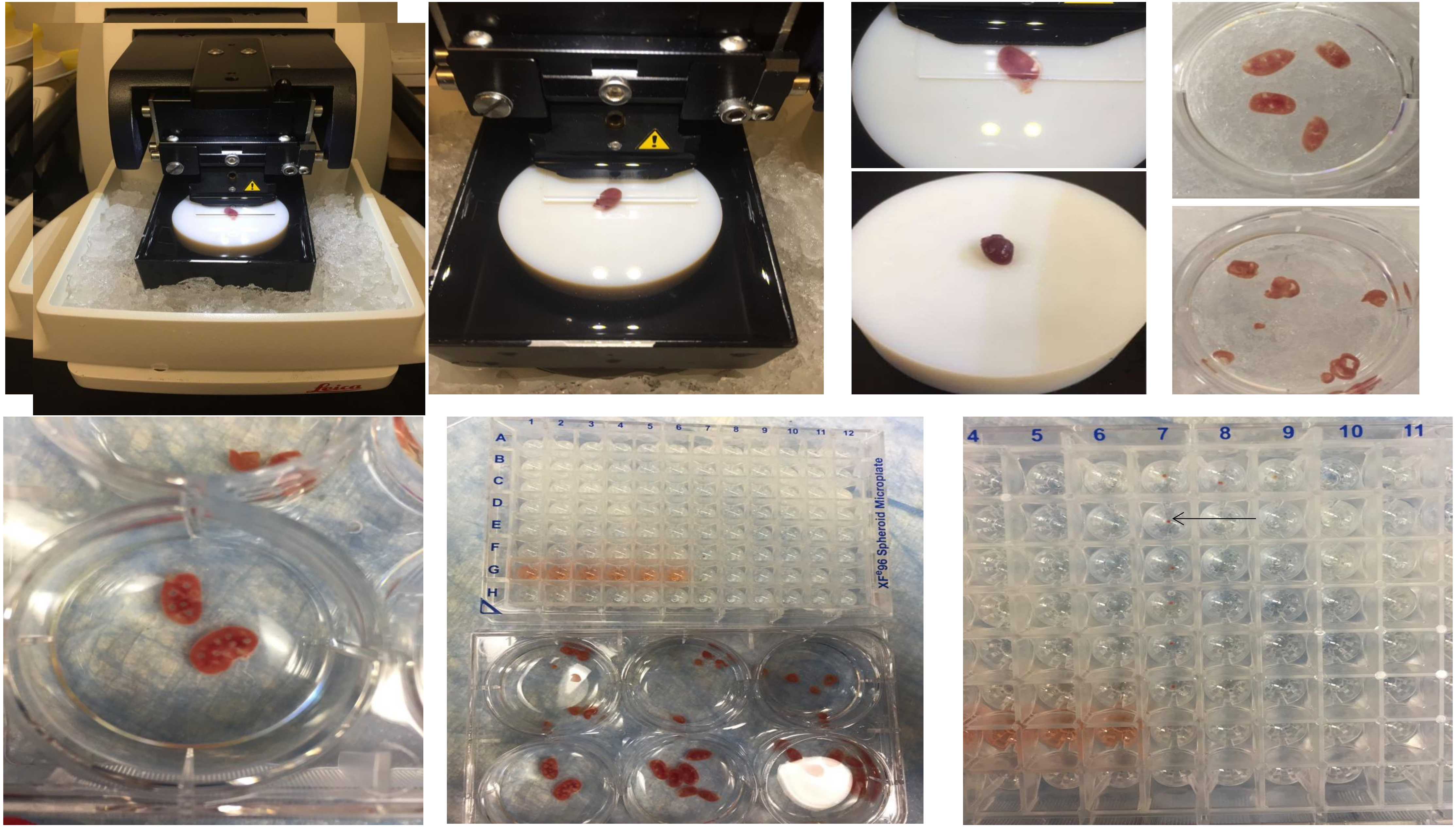
Leica
1 2 3 4 5 6 7 8 9 10 11 12
A B C D E F G H
XFe96 Spheroid Microplate
4 5 6 7 8 9 10 11

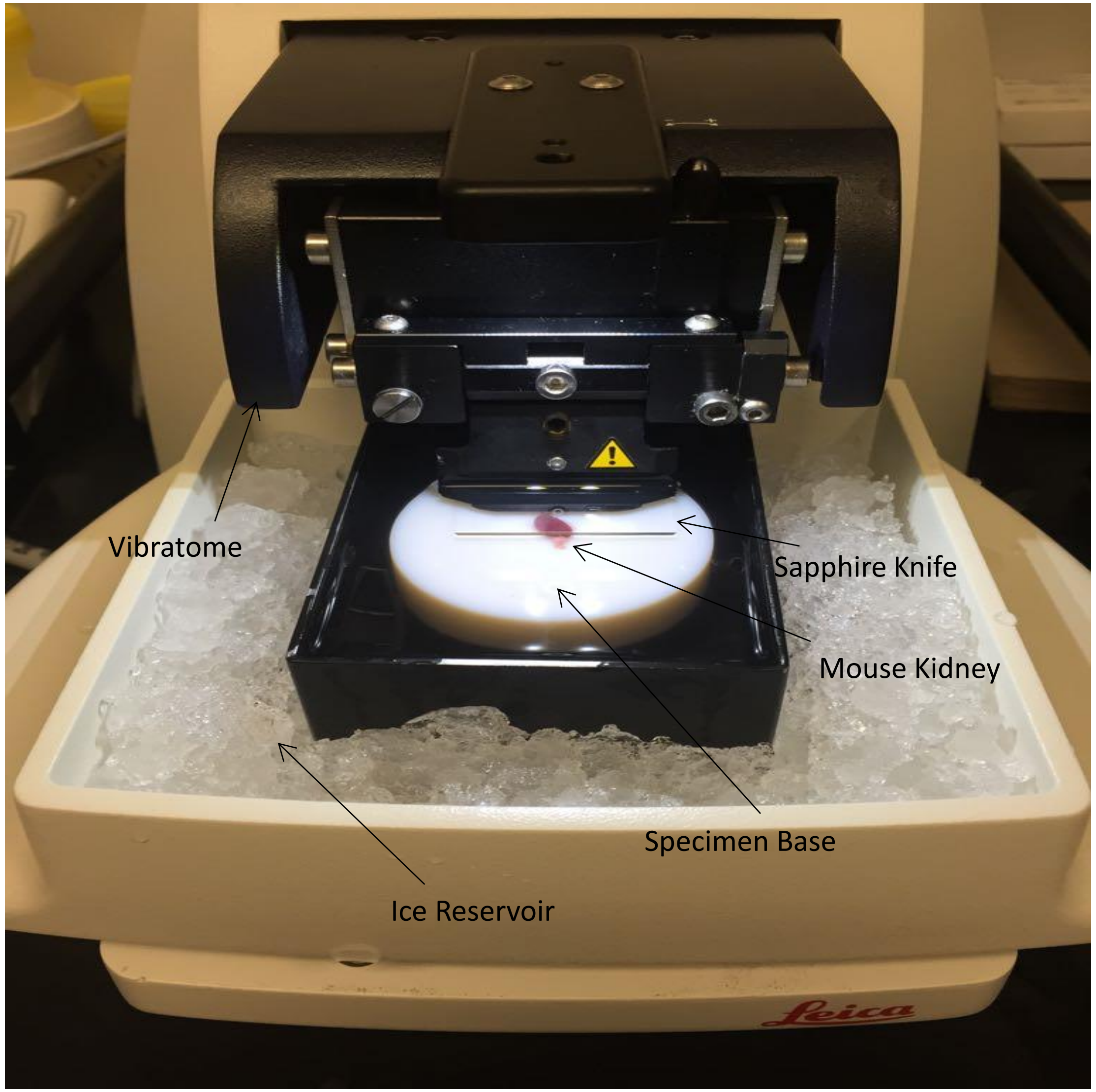
Vibratome
Sapphire Knife
Mouse Kidney
Specimen Base
Ice Reservoir
Leica
